# Effect of cold atmospheric plasma on common oral pathogenic microorganisms: a narrative review

**DOI:** 10.1080/07853890.2025.2457518

**Published:** 2025-01-27

**Authors:** Jiajun Xin, Hao Zhang, Yushen Li, Yifei Dai, Xiantao Chen, Jiatong Zou, Rui Wang, Zhihui Liu, Bowei Wang

**Affiliations:** ^a^ Department of Prosthodontics, Hospital of Stomatology, Jilin University, Changchun, People’s Republic of China; ^b^ Jilin Provincial Key Laboratory of Tooth Development and Bone Remodeling, Changchun, People’s Republic of China; ^c^ Department of Obstetrics and Gynecology, The Second Hospital of Jilin University, Changchun, People’s Republic of China

**Keywords:** Cold atmospheric plasma, oral microorganisms, oral infectious disease, antimicrobial, reactive oxygen and nitrogen species

## Abstract

**Background:**

The oral microbiota is a diverse and complex community that maintains a delicate balance. When this balance is disturbed, it can lead to acute and chronic infectious diseases such as dental caries and periodontitis, significantly affecting people’s quality of life. Developing a new antimicrobial strategy to deal with the increasing microbial variability and resistance is important. Cold atmospheric plasma (CAP), as the fourth state of matter, has gradually become a hot topic in the field of biomedicine due to its good antibacterial, anti-inflammatory, and anti-tumor capabilities. It is expected to become a major asset in the regulation of oral microbiota.

**Methods:**

We conducted a search in PubMed, Medline, and Wiley databases, focusing on studies related to CAP and oral pathogenic microorganisms. We explored the biological effects of CAP and summarized the antimicrobial mechanisms behind it.

**Results:**

Numerous articles have shown that CAP has a potent antimicrobial effect against common oral pathogens, including bacteria, fungi, and viruses, primarily due to the synergy of various factors, especially reactive oxygen and nitrogen species.

**Conclusions:**

CAP is effective against various oral pathogenic microorganisms, and it is anticipated to offer a new approach to treating oral infectious diseases. The future objective is to precisely adjust the parameters of CAP to ensure safety and efficacy, and subsequently develop a comprehensive CAP treatment protocol. Achieving this objective is crucial for the clinical application of CAP, and further research is necessary.

## 1. Introduction

Plasma is considered the fourth state of matter, alongside solid, liquid, and gas. It is made up of charged and neutral particles that result from the ionization of gases. These particles include ions, electrons, excited and ground state molecules, atoms, and ultraviolet photons. Despite being made up of charged particles, plasma is electrically neutral. When it comes into contact with gases and liquids containing nitrogen and oxygen, plasma also generates a large number of reactive oxygen and nitrogen species (RONS), such as hydroxyl radicals, hydrogen peroxide, superoxide anion radicals, singlet oxygen, nitrites, and nitrates. These reactive species play a significant role in the effects of plasma [1].

Plasma can be divided into two categories: high-temperature plasma and low-temperature plasma [2]. High-temperature plasma is a thermal equilibrium plasma, such as that found in stars and during nuclear fusion. In this type of plasma, the electrons and heavy particles reach the same temperature, which can soar up to 10^8 K. Creating high-temperature plasma in laboratory conditions is quite challenging. In contrast, low-temperature plasma is classified as a non-thermal equilibrium plasma and can be divided into thermal and non-thermal plasma. Recent advancements in science and technology have enabled the generation of non-thermal plasma at room temperature and pressure, known as cold atmospheric plasma (CAP) [3,4]. CAP has gained significant attention in the biomedical field due to its unique properties, which include low temperature and high reactivity. These characteristics make CAP safe for contact with the human body without causing damage [5,6]. The generation of CAP can come from various types of discharges, such as dielectric barrier discharge (DBD) and corona discharge. Regarding application, CAP delivered as a jet offers several advantages. This type of CAP can be maneuvered like a pen, allowing for precise delivery of the active ingredient and enhancing safety. However, when treating larger areas, continuous movement is often necessary to address its limitations [7].

In recent years, there has been a growing amount of research on CAP in the biomedical field. Table 1 presents some of the relevant studies. CAP technology can create coatings on biomaterial surfaces, enhancing their properties to meet clinical requirements [8,9,30]. Additionally, CAP shows promise in treating tumor diseases by inhibiting the growth of specific tumor cells through oxidative stress [10–12]. CAP also exhibits anti-inflammatory, antioxidant, and vascular regeneration-promoting abilities, contributing to treating skin diseases and wound healing [13–15]. Furthermore, with its ability to eliminate various pathogenic microorganisms, CAP is anticipated to be a new strategy for treating infectious diseases [16,17].

**Table 1.** Some recent research on plasma in biomedicine.

CAP carrier gas	CAP parameters	Results	References
Argon	300 A, 30 V, 40 L/min	A manganese-tricalcium phosphate coating was applied to titanium using arc plasma spraying. This coating enhanced the attachment and proliferation of NCTC L929 mouse fibroblasts but did not possess antimicrobial properties against *E. coli*, *E. faecium*, and *P. aeruginosa*.	[8]
Argon/Nitrogen	Argon: 100 scfh, Nitrogen: 10 scfh, 18–22 kW, 350A, 400A, 450A	Strontium, Magnesium, and Zinc coatings were successfully formed on the hydroxyapatite surface by vapor-induced pore-forming atmospheric plasma spraying. The coatings helped to promote osteoblast proliferation and inhibit *P. nigrescens*, *P. gingivalis*, and *F. nucleatum* adhesion.	[9]
Argon	4 kV, 40 kHz, 10 L/min	CAP can raise the level of ROS by targeting cancerous mitochondria, leading to swelling of the mitochondria. This promotes the activation of caspase-3, which triggers apoptosis in OSCC cells. Furthermore, CAP and cisplatin have been found to have synergistic effects, indicating that CAP is a potentially valuable complementary therapeutic option.	[10]
Air	≤14 kV, 40 mA, 20 kHz, 15W–25W	CAP treatment significantly reduced the cell viability and migration rate of SNU-449 and SNU-475 (the hepatocellular carcinoma cells) and induced apoptosis.	[11]
Air	6 kHz, 14 kV, 1μs, 1000 sccm	The plasma-activated medium produced by CAP treatment decreased the survival rate of ovarian cancer cells, demonstrating the potential of CAP in tumor cell treatment.	[12]
Helium	42 W, 220 V, 50 Hz, 3L/min	Treatment with CAP for 90 or 180 seconds considerably improved the healing of diabetic wounds in mice. This treatment led to a significant reduction in the expression of IL-6, TNF-α, iNOS, and SOD while also significantly increasing the levels of VEGF and TGF-β.	[13]
Air	39.87 kHz, 1.84V − 4.24V, 166.7 L/min	CAP treatment significantly reduced inflammation, promoted angiogenesis and fibroblast proliferation, and increased cellular antioxidant activity in type I diabetic rats.	[14]
Helium/Argon	0–10 kV, 20 kHz, 6–8 N/m^2^	CAP inhibited the growth of *P. aeruginosa*. It also decreased the expression of the virulence gene *alp*, reduced bacterial pathogenicity, and promoted the healing of burn wounds in mice.	[15]
Nitrogen	1.5 kpps, 0.5 atm	Respiratory syncytial virus can be effectively inactivated by 5 to 15 minutes of nitrogen plasma treatment.	[16]
Argon/Oxygen (999:1)	23W, 15L/min	*S. mutans* biofilms were incubated for 24 or 48 hours. Most of the bacteria in the biofilm were destroyed after 300 seconds of micro-plasma treatment.	[17]
Argon	10 W, 10 L/min	The 30-minute CAP treatment increased the surface energy of zirconia and did not change the roughness. *S. mutans* adhesion was significantly reduced; however, the biofilm thickness increased with time after treatment.	[18]
Argon	85 W, 13.56 MHz	A 15-minute CAP treatment improved the hydrophilicity of zirconium oxide, promoted the proliferation of osteoblasts, and inhibited the adhesion of *P. gingivalis*.	[19]
Oxygen/Argon	24 W, 100 Hz	A 12-minute treatment with either oxygen plasma or argon plasma improved hydrophilicity without altering the structure and roughness of the zirconia surface. Both plasmas enhanced osteoblast proliferation, with oxygen plasma being more effective.	[20]
Helium	2.85 kV, 17 kHz	After the helium CAP jet treatment, the surface morphology of zirconia remained unchanged. The treatment inhibited the growth of *S. mutans* and *P. gingivalis* and reduced biofilm formation. Zirconia abutments exhibited enhanced bacterial inhibition with increasing treatment time.	[21]
Argon/Oxygen (99:1)	300 W, 10 L/min	Adhesion of *P. gingivalis* was lowest immediately after plasma treatment and gradually increased with prolonged action time in the atmosphere.	[22]
Argon	500W, 500Hz, 20μs	Compared with pure titanium, the Ta_2_O_5_ coating pre-treated with PEO showed stronger antimicrobial properties against *Staphylococcus aureus* and *Actinobacillus actinomycetemcomitans*. It also promoted the proliferation of HSF and MG-63 cells. However, it was found to be inferior to Ta_2_O_5_ in terms of both antimicrobial ability and the ability to encourage the proliferation of soft tissue cells.	[23]
Argon/Hydrogen	600A, 65V, Argon: 40000 sccm, Hydrogen: 10000 sccm	The CeO_2_ coating was applied using the plasma spraying technique onto Ti-6Al-4V substrates. The resulting plasma-sprayed CeO_2_ coating exhibited enhanced proliferative and osteogenic activities in MC3T3-E1 and BMSC. Additionally, it showed stronger antimicrobial effects on *Enterococcus faecalis*, *Prevotella intermedia*, and *Porphyromonas gingivalis*.	[24]
Nitrogen	15 kV, 13 mA, 5 L/min	The CAP treatment enhances the adhesion strength to the core resin by chemically modifying the surface of the epoxy resin-based fiber post to increase the surface energy.	[25]
Oxygen/ Argon/ Carbon tetrafluoride	220V, 100W, 1 atm, 8.5 L/min	Argon and carbon tetrafluoride plasma enhanced the adhesion of porcelain zirconia through chemical modifications. Argon added oxygen elements, while carbon tetrafluoride appeared as oxyfluoride compounds. Oxygen plasma increased hydroxyl group implantation and raised porosity near the ceramic interface.	[26]
Ambient air	15 kV, 500 Hz	Through CAP treatment, a more adherent gelatin film loaded with Econazole can be obtained, allowing the drug to reside longer in the oral cavity without compromising its antifungal effect.	[27]
Argon/Helium	Argon: 1–2.5 L/min, Helium: 1–5 L/min	Plasma-treated alginate hydrogels can be used as storage carriers for RONS, which can help to achieve precise release and thus provide new ideas for cancer therapy.	[28]
Argon	1100 sccm	An injectable and shape-adaptable alginate PTH was synthesized by CAP treatment. It can release RONS within 30 minutes to kill osteosarcoma cells and induce the release of immunogenic signals to enhance their phagocytic uptake by dendritic cells.	[29]

Abbreviation: CAP, cold atmospheric plasma; sccm, standard cubic centimeter per minute; scfh, standard cubic feet per hour; ROS, reactive oxygen species; OSCC: oral squamous cell carcinoma; PEO: plasma electrolytic oxidation; RONS: reactive oxygen and nitrogen species; PTH: plasma-treated hydrogels.

In dentistry, plasma technology plays a significant role. CAP treatment has been found to increase the hydrophilicity of zirconia surfaces and promote osteoblast proliferation while inhibiting bacterial attachment [18–20]. One study demonstrated that using helium CAP jets to modify the surface of zirconia implant abutment materials significantly reduced the formation of *Streptococcus mutans* and *Porphyromonas gingivalis* biofilms without affecting the surface morphology [21]. The therapeutic effect is time-dependent, with a longer duration of action leading to a more pronounced effect. However, the inhibitory effect of CAP treatment on bacterial adhesion diminishes over time. For instance, on titanium dioxide surfaces treated with argon/oxygen CAP jets, the adhesion of *P. gingivalis* decreased immediately after treatment and then gradually increased over time [22]. Therefore, future studies should focus on the effective time frame after CAP treatment and explore ways to improve the persistence of bacterial inhibition. Additionally, plasma-related techniques can form a coating on the implant surface, enhancing its biocompatibility, antimicrobial and osteogenic properties and preventing peri-implantitis [23,24]. Some studies have utilized plasma polymerization to modify titanium implants, resulting in reduced adhesion of *Streptococcus sanguis* and *Ligilactobacillus salivarius* and improved biocompatibility [31,32]. CAP can enhance the bonding properties of dentin, various ceramic materials, and fiber posts [25,26,33]. Additionally, when combined with drug delivery systems, CAP treatment can produce a highly adhesive gelatin film loaded with drugs for topical oral drug delivery [27]. This approach overcomes challenges posed by humid environments and complex oral functions, allowing for effective long-term drug release and enriching the potential for clinical application. Canal’s team researched CAP-treated hydrogels and discovered that plasma treatment does not affect the ability of alginate to form hydrogels or alter its rheological properties. The study suggests that plasma-treated hydrogels can serve as carriers of RONS to release these species in a controlled manner to treat osteosarcoma, ovarian cancer, and other oncological diseases without invasive procedures [28,29,34].

**Figure 1.**
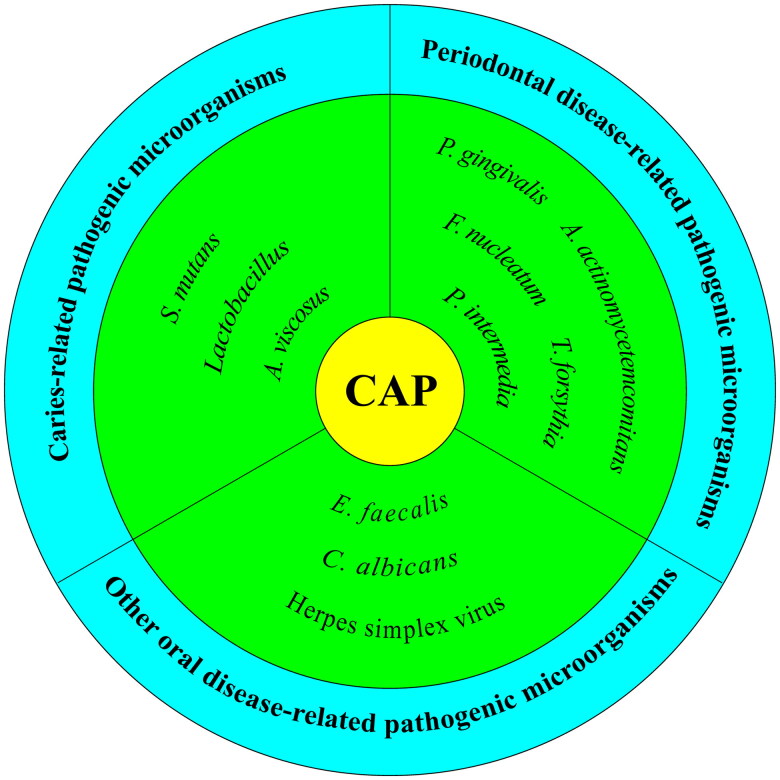
Microorganisms which the article will unfold from.

The oral microbiota is the second most complex microbial community after the gut microbiota. The host can tightly control the composition of the oral microbiota through cell-to-cell interactions or the release of potent antimicrobial substances. This is critical in the development of certain oral diseases. When the oral microbiota is disorganized, various oral infectious diseases and even oral cancer may develop [35–37]. Imbalances in the oral microbiota have been associated with systemic diseases such as gastrointestinal diseases, cardiovascular diseases, neurodegenerative diseases, and chronic obstructive pulmonary disease [38–40]. Recent studies have also shown that the oral microbiota may be linked to various cancers, such as genitourinary and colon cancers [41,42]. Therefore, preventing and treating oral pathogen infections is important. Current management tools for oral pathogenic microorganisms involve mechanical debridement with antibiotics as an adjunct. However, orally administered antibiotics are ineffective, and topically applied antibiotics are limited by the moist environment of the oral cavity and the complexity of the functions that are highly susceptible to loss [43,44]. Additionally, due to increased antibacterial resistance, there is an urgent need for a new method to eliminate oral pathogenic microorganisms effectively. This review will systematically summarize the research progress on the effects of CAP on common oral pathogenic microorganisms, as shown in Figure 1, discuss the antimicrobial mechanism of CAP, highlight the limitations of current research, and provide new ideas for treating oral infectious diseases.

## 2. CAP against caries-related pathogenic microorganisms

Caries is a chronic disease that destructively affects the hard tissues of teeth and is influenced by multiple factors. Currently, oral microorganisms are recognized as the primary contributors to this condition. When these microorganisms become imbalanced, they lead to rapid demineralization of dental hard tissues. Therefore, managing the microbiota associated with caries is essential for effective caries control. Table 2 shows some studies on CAP against caries-related pathogenic microorganisms.

**Table 2.** The effect of CAP against caries-related pathogenic microorganisms.

CAP carrier gas	CAP parameters	Results	References
Air	135W, 60 Hz, 18 m/s	Disrupting various microorganisms and reducing the microbial viability of 5-day-old *S. mutans* biofilms by 80%.	[45]
Argon	45 W, ∼30 kHz, 110 V, 8 L/min	Decreasing the biofilm activity of *S. mutans* on composite resin’s surface, affecting the metabolic state, damaging the biofilm structure, and promoting genetic damage.	[46]
Argon	8 W, 5000 sccm, 1.1MHz, 2–6 kVpp	Enhancing the biofilm resistance towards *S. mutans* of six disinfectants, including 0.1% CHX.	[47]
Argon	10 W	Processing for 1 minute reduced *S. mutans* biofilm on the surface of hydroxyapatite by about 90% and the bacterial reduction was further increased to more than 96% with the combined application of 0.2% CHX solution.	[48]
Argon	2.5 W, 2500 sccm	Combination with GNP further reduced *S. mutans* activity on the tooth surface to 5 logs; TEM observations showed that plasma treatment caused only cell wall perforation, whereas the combination of 30nm GNP treatment resulted in significant cell rupture.	[49]
Argon	10 W, 2000 sccm	Less than 15 seconds of exposure time completely kills *S. mutans* on filter paper, slides, and PTFE films. Under the same conditions, *L. acidophilus* was inactivated in 5 minutes.	[50]
Helium/Oxygen/ Nitrogen	2.45 GHz, 2.5 W Helium: 2L/min Oxygen: 1.2L/min Nitrogen: 1.5L/min	On agar plates, *Lactobacillus casei* growth was completely inhibited. On dentin sections, a 4-log reduction was achieved.	[51]
Argon	3000 sccm, 3.0 W, 6.0 mA, 0.5 kV	On the hydroxyapatite disk, a plasma exposure time of 10 seconds kills all *L. acidophilus* at low concentrations (2.1 × 10^8^–2.4 × 10^8^ cfu/mL). For the medium inoculum concentration (9.8 × 10^8^–2.4 × 10^9^ cfu/mL), it took 13 seconds. High inoculum concentration (1.7 × 10^9^–3.5 × 10^10^ cfu/mL) resulted in only a 1.5–2 log reduction of *L. acidophilus*.	[52]
Oxygen/Nitrogen/ Argon	5 L/min	Except for argon, CAP had a significant bactericidal effect when irradiated for 60 seconds at a distance of 20 mm from the culture medium. The inhibitory effect of CAP on *A. actinomycetemcomitans* was more pronounced than on *L. fermentum* and *S. mutans*.	[53]
Argon/Oxygen (98:2)	18 kV, 10 kHz, 5 L/min	PAW resulted in nearly a 5-log reduction in colony-forming units for *P. gingivalis*, *A. viscosus*, and *S. mutans* by 20, 40, and 60 seconds of treatment time, respectively.	[54]

Abbreviation: CAP, cold atmospheric plasma; CHX, chlorhexidine; GNP, gold nanoparticles; sccm, standard cubic centimeter per minute; PTFE, polytetrafluoroethylene; PAW, plasma-activated water; cfu, colony forming units.

### 2.1. Streptococcus mutans

*S. mutans* is a type of bacteria commonly found in dental plaque biofilms and a major contributor to tooth decay [55]. This bacterium produces enzymes known as glucosyltransferases (GTFs), which utilize sucrose to create a variety of polymers that form the main components of the biofilm matrix. *S. mutans* also possesses high-affinity adhesins on its surface, allowing it to colonize teeth without sucrose. Additionally, *S. mutans* metabolizes carbohydrates to produce organic acids, significantly acidifying the environment. However, the bacteria have mechanisms to resist the harmful effects of this acidity [56]. Combining these factors makes *S. mutans* a significant cause of tooth decay, highlighting its importance as a target for early caries control [57].

According to Duarte et al. a good bactericidal effect on *S. mutans* can be achieved by using an air plasma jet [45]. The study found that the growth of *S. mutans* was inhibited by the treatment, and the viability of the bacteria in the biofilm was reduced by up to 80%. The effect of the treatment increased as the treatment was prolonged. These findings were also confirmed by Nima et al. [46]. In their experiments, *S. mutans* biofilm on a resin disk was treated with argon CAP for varying durations. The results showed that the colony-forming units decreased significantly, and the longer the treatment time, the more significant the effect. The SEM observation revealed that the biofilm structure was damaged by the CAP treatment, while the PCR results showed that the DNA structure was also damaged.

Several researchers have investigated the combined application of CAP with other antimicrobial methods to improve its performance. Koban et al. [47] combined argon CAP jets with disinfectants such as 0.1% chlorhexidine (CHX), 0.1% octenidine (OCT), 0.1% polyhexanide (PHMB), 0.6% sodium hypochlorite (NaOCl), 1.5% hydrogen peroxide (H_2_O_2_), and 20% ethylenediaminetetraacetic acid (EDTA). They discovered that the synergistic effect of the jet with these disinfectants significantly enhanced the effectiveness of the anti-*S. mutans* biofilm. Hong et al. found that argon CAP reduced bacteria by about 90% after 1 min of treatment of *S. mutans* biofilms on hydroxyapatite surfaces [48]. When they combined the argon CAP with a 0.2% CHX solution, the bacterial reduction rate increased to more than 96% with a better antibacterial effect.

Plasma has also been combined with gold nanoparticles (GNP) to enhance its antimicrobial effects. Park et al. found that the argon CAP jet effectively killed *S. mutans* on slides, resulting in a 5-log reduction in activity [49]. However, only a 3-log reduction was shown when treating bacteria on dental surfaces. Combination with GNP followed by treatment resulted in a 5-log reduction in bacterial activity on the dental surfaces, while GNP alone did not show any bactericidal effect. TEM observations showed that only cell wall perforation was caused by plasma treatment, while combined GNP treatment resulted in significant cell rupture, further improving the killing effect of *S. mutans*. In recent years, some researchers have combined herbal medicines with low-temperature plasma. Park et al. [58] combined licorice extract with low-temperature plasma and found that the co-treatment resulted in good bacterial inhibition of *S. mutans*.

However, the safety of direct plasma irradiation has not been established, and it often takes time to kill bacteria below the detection limit. Therefore, plasma-activated water (PAW), an indirect application method, has been developed. It has a similar bactericidal activity to direct plasma irradiation and was obtained by exposing distilled water to a CAP environment [54]. In a study by Tasaki et al. dentin infection models were prepared on extracted teeth, and bacteria were recovered from depths of 0.8, 1.6, and 2.4 mm and subsequently cultured. Bacterial counts dropped significantly after only 10 s of PAW exposure [59]. Another study by Qiao et al. also affirmed the antibacterial ability of PAW [60]. Additionally, PAW has shown significant benefits in tooth whitening, wound healing, periodontitis treatment, and various other areas of research potential [61].

Overall, CAP has shown a good inhibitory effect on *S. mutans*, and when used with other antibacterial agents, its effectiveness is further increased. The use of PAW has also demonstrated similar bactericidal effects on *S. mutans*. Therefore, CAP may be beneficial in the prevention and early treatment of caries in clinical practice.

### 2.2. Lactobacillus

*Lactobacillus* is a type of bacteria known for its ability to break down sugars to produce acidic byproducts quickly. It can thrive in acidic and low-pH environments, making it a significant factor in the development of dentin caries [40,62]. Research has shown that *Lactobacillus* is more prevalent in dental plaque of individuals with active caries than those without and is often used as a microbial marker for progressive caries risk [63]; (Kong et al. 2022).

There are limited studies on the effects of CAP on *Lactobacillus*, but the ones available confirm its good inhibitory effect. Yang et al. conducted experiments on filter paper, slides, and polytetrafluoroethylene films and found that argon plasma jets completely killed *S. mutans* in less than 15 s of exposure [50]. However, it took nearly 5 min to achieve consistent results for *Lactobacillus acidophilus*, which may be due to the size and structure of the two bacteria. The experiment confirmed the effectiveness of CAP against *L. acidophilus*. In the experiments of Rupf et al. the growth of *Escherichia coli* and *Lactobacillus casei* in the irradiated areas of agar plates was entirely inhibited by CAP, and the inhibitory effect on *C. albicans* and *S. mutans* increased with increasing treatment time [51]. Furthermore, the treatment of dentin sections reduced the number of these microorganisms by 4 logs or more. This indicates that CAP could be used as a novel disinfection method to contribute to dental disinfection.

Blumhagen et al. conducted a study to investigate the effect of argon CAP on different concentrations of oral bacteria [52]. They inoculated hydroxyapatite disks with *Lactobacillus acidophilus* at various initial concentrations and then examined the changes in bacterial morphology after plasma exposure using SEM. The study found that *L. acidophilus* at low and medium inoculum concentrations were entirely killed by 10s and 13s of plasma exposure, respectively. However, *L. acidophilus* was reduced by only 1.5–2 logs at high inoculum concentrations. The inactivation mainly occurred in the top bacteria, and the shadowing effect that produced bacterial debris reduced the inactivation of the bottom bacteria. This is similar to the effect of CAP on biofilm penetration, which will be explained later in the text. Abonti et al. investigated the effect of CAP jets generated by different gases on *Lactobacillus fermentum* [53]. The carrier gas types used in the study were oxygen, nitrogen, argon, and a 1:1 mixture of oxygen/nitrogen. All of these plasma jets were effective against *L. fermentum* on agar plates, and the most effective plasma gas was oxygen. This may be associated with more reactive oxygen species (ROS) production.

The current study indicates that measuring CAP’s effect on *Lactobacillus* is inadequate. Additional experimental studies are necessary to strengthen the findings. This will provide a more robust theoretical foundation for using CAP in treating progressive caries.

### 2.3. Actinomyces viscosus

*A. viscosus* is a harmful pathogen that causes root caries, and it can be found in almost all root caries [64]. The hyphae on its surface can stick firmly to α1-type collagen in the dentin, creating a fenestrated structure that accelerates plaque formation. This bacterium can also produce acid under various growth conditions [65]. Recent research has shown that *Actinomyces* genes are expressed at similar levels in both sound and carious root biofilms. These bacteria can live symbiotically on root surfaces, but they cause caries due to their ability to survive in acidic environments and metabolize sugars [66].

Li et al. obtained plasma-activated water (PAW) by treating 10 ml of sterile distilled water with a mixture of 98:2 argon and oxygen as the carrier gas for CAP for 20 min [54]. They then examined the effects of PAW on *P. gingivalis*, *A. viscosus*, and *S. mutans in vitro*. The results showed that after 20 s of treatment with PAW, the CFU of *P. gingivalis* reduced by nearly 5 logs. However, *A. viscosus* and *S. mutans* required 40 and 60 s, respectively, to achieve a similar effect. This indicates that Gram-negative bacteria are more susceptible to the bactericidal effect of plasma-activated water than Gram-positive bacteria. Additionally, *A. viscosus* is more susceptible to the impact of PAW than *S. mutans*. This finding can be explained by the thickness of the cell wall, which is consistent with the results of a previous study conducted by Abonti et al. [53].

Although there are few studies on the effect of CAP on *A. viscosus*, the research so far suggests that it has an inhibitory effect on these bacteria. However, the actual antibacterial impact and mechanism of action need further exploration and refinement.

In conclusion, CAP has a promising inhibitory effect on many microorganisms that cause caries. This suggests that it could be used as a new approach for preventing and treating early-stage caries. While research is still to maximize its anti-caries function and put it into clinical application, the future looks positive.

## 3. CAP against periodontal disease-related pathogenic microorganisms

Periodontitis is a significant cause of tooth loss and is linked to several systemic diseases. Dental plaque is considered the most important factor contributing to this condition. Currently, periodontitis is primarily treated through mechanical debridement accompanied by topical antibiotics. However, invasive treatment methods often lead to adverse patient experiences, and the rise in antibiotic resistance has reduced treatment efficacy. Therefore, there is a pressing need for innovative treatments for periodontitis. Table 3 shows some studies on CAP against periodontal disease-related pathogenic microorganisms.

**Table 3.** The effect of CAP against periodontal disease-related pathogenic microorganisms.

CAP carrier gas	CAP parameters	Results	References
Helium/Oxygen	10 kHz, 1.6μs, 8 kV	An in vitro study found that a 5-minute treatment caused almost total bacterial death in *P. gingivalis* biofilms. An in vivo study in male rabbits found that a 10-minute CAP treatment did not result in pathologic changes in their oral mucosa.	[67]
Helium	31 kHz, 10 kV, 0.6 W, 1000 sccm	In two-day HW24D-1 biofilms, both one to seven-minute treatments reduced bacterial viability in a time-dependent manner. HGF cells without CAP treatment and HGF cells treated with one to seven minutes had similar DNA indices.	[68]
Argon	50 W, 5000 sccm	After being subjected to CAP treatment for 1–3 minutes, W83 biofilm CFU was significantly reduced. There was no significant difference between the two treatments, both of which had lower efficacy than 0.2% CHX. In both cases, low cytotoxicity was observed in the gingival epithelium with high cell viability. The 3-minute treatment was found to be more cytotoxic than the 1-minute treatment.	[69]
Helium	10 kHz, 7 kV, 5000 sccm	After 3 minutes of treatment, *P. gingivalis* biofilm CFU on sandblasted and acid-etched titanium discs were significantly lower than those of the helium group and below the threshold level of detection after 5 minutes. A mixture of live and dead bacteria was visible in the marginal areas after 3 minutes of treatment, and after 5 minutes, most of the remaining bacteria were dead.	[70]
Air	200 Hz, 1 L/min	After a treatment of 3 minutes, the number of *S. mutans* bacteria decreased by more than 4 logs and was almost completely eliminated after 5 minutes. *P. gingivalis* was completely eliminated after 1 minute of treatment, and the killing rates were not affected by the distance of the treatment. The number of viable *Enterococcus faecalis* was significantly reduced through CAP treatment in a time-dependent manner, with complete elimination observed within 7 minutes of treatment.	[71]
Argon/ Oxygen	6 mA, Argon: 3000 sccm Oxygen: 30 sccm	For 3-day *P. gingivalis* biofilms, argon plasma treatment for 1, 2, and 5 min reduced the bacterial load by 76.97 ± 1.21%, 75.09 ± 1.80%, and 71.95 ± 4.41% (p < 0.05), respectively. The addition of 1% oxygen reduced the bacterial load by 79.62 ± 1.57%, 80.02 ± 1.78%, and 83.38 ± 2.25% for 1, 2, and 5 min, respectively (p < 0.05).	[72]
Oxygen/Nitrogen/ Argon	5 L/min	Except for argon, CAP had a significant bactericidal effect when irradiated for 60 seconds at a distance of 20 mm from the culture medium. Among them, oxygen CAP was the most effective, resulting in zero survival of the accompanying *A. actinomycetemcomitans.* The inhibitory effect of CAP on *A. actinomycetemcomitans* was more pronounced than on *L. fermentum* and *S. mutans*.	[53]
Air(DBD)	DBD-Rod: 15.4 kV, 40 kHz 250μs, 1000 Hz DBD-Plate: 12 kV, 20 kHz 4000μs, 100 Hz	Before contamination with *A.actinomycetemcomitans* and *S. mutans*, CAP treatment of soft reline palatal occluders resulted in the CFU reduction of approximately 2 logs. For early biofilms (90 minutes), treatment resulted in at least a 3-log reduction in CFU and a 40–60% loss of bacterial viability. For late biofilms (24 hours), treatment resulted in a 2-log reduction in CFU and a 20–40% loss of bacterial viability.	[73]
Ambient air	50 kHz, 8 W	The 30-second treatment completely killed *F. nucleatum* and *P. intermedia*, and *T. forsythia* was completely killed within 60 seconds. CAP treatment of dentin specimens did not significantly reduce the amount of biomass covering the bacterial and biofilm matrices, but the metabolic activity was reduced by approximately 70% after 60 seconds of treatment and by approximately 95% after 120 seconds.	[74]
Helium / Oxygen (999:1)	8 kV, 8 kHz, 1.6μs, 2 L/min	Significantly lower numbers of *P. gingivalis and T. forsythia* were found in Beagles with additional CAP treatment compared to conventional mechanical scraping and medicated rinsing for peri-implantitis.	[75]

Abbreviation: CAP, cold atmospheric plasma; CHX, chlorhexidine; sccm, standard cubic centimeter per minute; CFU, colony forming units; HGF, human gingival fibroblasts; DBD, dielectric barrier discharge.

### 3.1. Porphyromonas gingivalis

*P. gingivalis* is an anaerobic bacterium that is classified as Gram-negative. It is a part of the “Red Bacterial Complex” and is closely linked to advanced periodontal lesions [76]. This bacterium invades the periodontal tissues locally, dodges host defense mechanisms, and utilizes various virulence factors that lead to dysregulation of the inflammatory response [77]. *P. gingivalis* relies on various substances, such as gingipains, adhesins, lipopolysaccharides, cysteine proteases, and others, to promote its growth and virulence [78].

CAP has been found to have a similar effect on *P. gingivalis* as it does on *S. mutans*. Studies have shown that CAP treatment can effectively kill the bacteria in *P. gingivalis* biofilms. Liu et al. conducted an *in vitro* study and found that almost all bacteria in the CAP-treated group died, while most of the control group survived [67]. They also conducted an *in vivo* study on male rabbits and found that CAP did not cause any pathological changes in the normal mucosa, confirming the biological safety of CAP.

In recent years, Lima et al. have shown that a helium CAP jet significantly decreased the viability of bacteria in 2-day-old *P. gingivalis* HW24D-1 biofilms in a time-dependent manner [68]. Another study by Carreiro et al. cultured *P. gingivalis* W83 biofilms on titanium discs for 5 days. They found that the colony-forming units decreased after CAP treatment but not as significantly as after 0.2% CHX treatment [69]. Interestingly, the study also found that different CAP treatment times did not show significant differences. Moreover, the study confirmed the safety of CAP treatment *in vitro* and found that it was not genotoxic to gingival epithelial cells and fibroblasts. Additionally, CAP was found safe for the gingival epithelium and did not cause any tissue damage *in vivo*. Therefore, cold atmospheric plasma is expected to be an adjunctive therapy for periodontal disease treatment [79].

The decontaminating effect of different treatment times and distances of CAP has been studied. A study by Lee et al. used a helium CAP jet to treat *P. gingivalis* biofilms on titanium discs and grouped them according to the treatment time [70]. They confirmed that the jet had a good destructive effect on *P. gingivalis* biofilms with a time-dependent effect. Another study by Hirano et al. found that *P. gingivalis* was killed entirely by a 1-minute CAP treatment, regardless of the treatment distance [71]. Additionally, adding a small amount of oxygen to the inert gas increases the bactericidal effect of CAP. Hong et al. treated *P. gingivalis* biofilms of different growth times with argon CAP jets with or without 1% oxygen and found that the plasma with 1% oxygen had a more pronounced bactericidal effect [72]. They also demonstrated that CAP-treated *P. gingivalis* biofilms were less resistant to amoxicillin and more sensitive to H_2_O_2_. This suggests that CAP treatment enhances biofilm susceptibility to host defenses and improves drug efficacy. Therefore, CAP pretreatment before conventional drug therapy may change the course of treatment for periodontitis and other infectious diseases.

This indicates that CAP, a novel antimicrobial tool, exhibits a strong antibacterial effect against *P. gingivalis*. In future periodontitis treatments, CAP is anticipated to serve as an adjunctive option to enhance bacterial clearance.

### 3.2. Aggregatibacter actinomycetemcomitans

*A. actinomycetemcomitans* is a harmful bacterium known to cause aggressive periodontitis, particularly limited aggressive periodontitis, and is also linked to chronic periodontitis [80]. This bacterium attaches to the periodontal tissues *via* hyphae and adhesins and then releases endotoxins and exotoxins that cause damage to the periodontal tissue [81]. Leukotoxin, in particular, plays a crucial role in this process by interacting with various inflammatory cells, leading to cell lysis and the release of large amounts of pro-inflammatory factors, which ultimately causes inflammation of the periodontal tissues [82].

Abonti et al. conducted a study to investigate the effect of different CAP jet working gases on *A. actinomycetemcomitans* [53]. The study found that all plasmas, except for argon CAP, irradiated at 20 mm from the medium for 60 s showed a significant bactericidal effect. The jet with oxygen CAP was the most effective, with *A. actinomycetemcomitans* showing zero survival. This could be because of the higher levels of ROS production. Interestingly, the study found that 20 mm was more effective than 2 mm when comparing the effect of distance. This may seem counterintuitive, which may be because the proper distance confers the spatial and temporal conditions needed to produce the active substance. Moreover, the study found that CAP had a more significant inhibitory effect on *A. actinomycetemcomitans* than other bacteria, such as *Lactobacillus fermentum* and *S. mutans*. This is probably because Gram-negative bacteria, like *A. actinomycetemcomitans*, are more sensitive to ROS due to their thinner peptidoglycan layer. Mai-Prochnow et al. found a similar phenomenon, suggesting that CAP inactivation is related to the thickness of the bacterial cell wall, which will be further elaborated later [83].

Liguori et al. conducted a study investigating the impact of two DBD-type CAPs on biofilm adhesion and survivability [73]. The researchers found that treating soft reline palatal obturators with CAPs before contamination with *A. actinomycetemcomitans* and *S. mutans* effectively prevented the adhesion of both bacteria. When they used CAPs to treat palatal obturators that had already formed biofilms, the survival of the bacteria was significantly reduced. This was especially true for early biofilms, which saw a 40–60% reduction in bacterial survival and a 3-log reduction in CFU. The study also confirmed that the treatment did not lead to significant changes in the overall mechanical properties of the occluder and was non-toxic to human primary cells.

The impact of CAP on *A. actinomycetemcomitans* is significant, not only in killing the bacteria but also in inhibiting its adhesion to surfaces. From the available information, the killing effect of CAP on *A. actinomycetemcomitans* is even stronger than that on *S. mutans*, which has been studied more frequently. However, the study of CAP on *A. actinomycetemcomitans* is relatively small, and more specific conclusions are yet to be explored. Hopefully, subsequent studies will further clarify the issue.

### 3.3. Other periodontal pathogens

Various microorganisms such as *Fusobacterium nucleatum*, *Tannerella forsythia*, and *Prevotella intermedia* are closely associated with periodontal disease. *F. nucleatum* has virulence factors such as adhesins that promote adhesion and colonization, lipopolysaccharides that stimulate inflammation and bone resorption, and serine proteases that obtain nutrients [84]. It is part of the “orange bacterial complex” that supports colonization by the “red complex” and is essential for the progression of periodontitis [85]. In addition, synergistic interactions with *P. gingivalis* will further exacerbate periodontal tissue inflammation and bone loss [86]. *T. forsythia* belongs to the red complex, a crucial pathogen in periodontitis. Its surface proteins mediate bacterial binding to oral epithelial cells, associated with alveolar bone loss [87]. Enolase, a glycolytic enzyme, binds to fibrinogen and increases the expression of proinflammatory factors, and karilysin, a metalloprotease, upregulates soluble TNF-α concentrations and inhibits the complement pathways [88,89]. Furthermore, the synergistic effect of *T. forsythia* GroEL with IL-17 promotes periodontal inflammation and bone resorption [90]. *P. intermedia* is often found in patients with chronic periodontitis and acute necrotizing ulcerative gingivitis [91]. It is also a dominant bacterium in gingivitis during pregnancy, probably due to altered hormone levels, which allows it to consume estrogen and progesterone as a source of nutrients instead of vitamin K [92]. Its hyphae, endotoxin, and secreted protein hydrolyzing enzymes are all virulence factors contributing to periodontal disease [93].

Jungbauer et al. treated three different bacteria using piezoelectric direct discharge CAP [74]. The treatment completely killed two of the bacteria, *F. nucleatum* and *P. intermedia*, within 30 s. *T. forsythia,* which did not decrease significantly for 30 s, was also wholly killed within 60 s. Subsequently, the researchers investigated the effect of CAP on various biofilms. They found that while CAP application did not significantly reduce the biomass covering bacteria and biofilm substrates, it decreased metabolic activity by approximately 70% after 60 s and by approximately 95% after 120 s of treatment. Furthermore, these treatments did not affect the subsequent biofilm formation on dentin or titanium specimens. This suggests that CAP can be used as an adjunct to periodontitis treatment. Shi et al. induced peri-implantitis in Beagles by ligation [75]. After three months, animals that were additionally treated with 0.1% oxygen/helium CAP jets had significantly lower counts of *P. gingivalis* and *T. forsythia* and significantly higher bone levels compared to the simple treatment group, which only received mechanical scraping with CHX rinsing.

It can be concluded that CAP has a positive impact on inhibiting various pathogens related to periodontal diseases. Additionally, it can aid in the healing of periodontal tissues and regulate oxidative stress [94–96]. Therefore, CAP has the potential to become a new treatment option for periodontal diseases and peri-implantitis. However, it is important to note that most of the studies on CAP have been conducted *in vitro*. Therefore, it is necessary to determine how to adjust CAP parameters to balance biological safety and clinical effectiveness. Conducting clinical studies to apply the findings to practical use is also crucial and requires extensive research.

## 4. CAP against other oral disease-related pathogenic microorganisms

Besides caries and periodontal disease, CAP also effectively inhibits microorganisms linked to oral diseases, including apical periodontitis. Table 4 shows some studies on CAP against other oral disease-related pathogenic organisms.

**Table 4.** The effect of CAP against other oral disease-related pathogenic microorganisms.

CAP carrier gas	CAP parameters	Results	References
Ambient air	3.5 kV, 4.0 kHz, 0.5-1 W	Treatment for 1min reduced *E. faecalis* CFU on agar plates by 7 logs, 3–5min by 8 logs, and 10min by 9 logs. Treatment for 5 minutes resulted in a 3-log reduction in *E. faecalis* biofilm CFU and a 5-log reduction for 10min. This effect was comparable to 0.2% CHX.	[97]
Helium/Oxygen (98:2)	10 kV, 10 kHz, 1.6 μs, 1 L/min	The 10-minute CAP treatment resulted in almost total elimination of *E. faecalis* colonies and biofilm for 48 hours. Residual colonies after treatment, with disrupted structures and sparse matrix-like material, had 100 times fewer *E. faecalis* compared to the control group.	[98]
Air	2.6 kHz, 7.6 mJ 1.1 W	DBD-type CAP treatment reduced *E. faecalis* by 90% after 2 minutes and increased with time.	[99]
Argon/Oxygen (98:2)	18 kV, 10 kHz, 5 L/min	For *E. faecalis* biofilm in root canals, CAP treatment for 2–6 minutes was not as effective as conventional calcium hydroxide. When treated for 8–10 minutes, then the antimicrobial effect was superior to calcium hydroxide, and no residual *E. faecalis* was detected in the samples after 10 minutes of treatment.	[100]
Helium/Oxygen (50:2)	16 kV, 10 kHz, Helium: 5 L/min, Oxygen: 0.2 L/min	CAP was superior to sodium hypochlorite for sterilization in the middle third of the root canal, while there was no significant difference between the coronal and apical third. CAP killed *E. faecalis* better than ozone.	[101]
Argon	4800 sccm	For *E. faecalis* inoculated in root canals, the combination of CAP and 2% CHX was most effective in the whole layer of root canals at the depth of 800 μm, as well as in the superficial layer of root canals from 0–300 μm. The difference was insignificant at a depth of 300–500 μm, while at a depth of 500–800 μm, CHX applied alone was the most effective.	[102]
Argon or Argon/Oxygen (99:1)	5000 sccm	After 6 minutes of treatment, neither CAP was found to be as significant as 3% NaOCl against *E. faecalis*. Extending the application time of CAP containing 1% oxygen further reduced the density of *E. faecalis*, whereas extending the application time of NaOCl or argon CAP showed no significant change. After 12 min of treatment, NaOCl combined with CAP containing 1% oxygen showed the best disinfecting effect.	[103]
Helium/ Oxygen	8 kV, 8 kHz, 1.6μs, Helium: 1 L/min, Oxygen: 0.01 L/min	Both CHX and CAP treatments alone significantly increased the number of dead bacteria in *E. faecalis* biofilms and multispecies biofilms on dentin disks, with comparable effects. The bactericidal effect of the CHX-modified CAP jet was even more significant. The number of dead bacteria in the 1-week biofilm was significantly higher than in the 3-week biofilm.	[104]
Helium	8 kV, 8 kHz, 1.6μs, 2 L/min	The flow of helium through 3% hydrogen peroxide and the subsequent plasma jet significantly reduced the number of *E. faecalis* biofilm colonies in extracted anterior teeth root canals. The reduction was measured at 6.237 logs in 2 minutes and 7.027 logs in 4 minutes, which was found to be more effective than the conventional CAP treatment.	[105]
Ambient air	9 kV, 2 kHz	For *E. faecalis* biofilms, there was a synergistic effect between CAP and EDTA, and between CAP and TSC.	[106]
Helium or Helium/ Oxygen	5 kV, 25 kHz Helium: 4000 sccm, Oxygen: 20 sccm	For *E. faecalis* biofilms inoculated in root canals, 8-minute helium plasma treatment was as effective as PDT, whereas helium plasma containing 0.5% oxygen showed the best results. SEM showed that after PDT treatment, the biofilm structure on the root canal surface was disrupted and the dentin tubule openings were almost detected, whereas after an 8-minute helium plasma treatment containing oxygen, the biofilm disappeared from the surface of the root canals and the dentin tubule openings were completely detected.	[107]
CAP jet: Argon DBD: Ambient air	CAP jet: 4300 sccm DBD: 10 kV, 300Hz 10μs, 450mW	Of all the treatments, NaOCl was most effective in killing *E. faecalis* biofilm. In the crown side of the root canal, PDT and CAP jet were equally effective and both were significantly better than DBD CAP.	[108]
Argon/ Oxygen (98:2)	18 kV, 10 kHz, 5 L/min	A 3-day-old biofilm of *E. faecalis* in the root canal was treated with CAP for 3-12 minutes. As a result, the CFU count gradually decreased to 0. The treatment had no significant effect on the microhardness and roughness of the pulpal dentin.	[109]
Helium	10 kV, 10 kHz, 2 L/min	Helium CAP was found to be comparable to 2% CHX in killing *E. faecalis* through in vivo and in vitro experiments, and Helium CAP modified by 2% CHX solution showed higher antimicrobial efficacy. Significant increase in BV/TV and decrease in inflammatory cells and bacterial residues after treatment.	[110]
Helium/Oxygen/ Nitrogen	2.45 GHz, 2.5 W, Helium: 2 L/min, Oxygen: 1.2 L/min, Nitrogen: 1.5 L/min	*C. albicans* attached to dentin sections decreased by more than 4 log CFU after 6 min of treatment, and almost complete killing of *C. albicans* was achieved after an extended period.	[51]
Helium	10 kV, 10 kHz, 2000 sccm	A 15-second treatment demonstrated 90% effectiveness in killing *C. albicans*, and over 30 seconds demonstrated 99%.	[111]
Air	10 L/min	The duration of CAP treatment inversely affected the number of viable *C. albicans*; a 20-second treatment resulted in almost 90% loss, while a 55-second treatment reduced the number below the detection limit.	[112]
Helium	1.8 W, 2000 or 4000 sccm	The flow rate of 2000 sccm versus 4000 sccm did not significantly affect the size of *C. albicans* inhibition zones, but the morphology of the inhibition zones was more irregular at 4000 sccm compared to 2000 sccm. The size of *C. albicans* inhibition zone decreased with the change of treatment distance from 2 cm to 3 cm.	[113]
Argon/Oxygen (98:2)	10 kHz, 18 kV, 5 L/min	Following the use of CAP treatment, there was no notable variance in the surface roughness, flexural strength, and elasticity modulus of the acrylic resin. However, there was a significant decrease in the contact angle, a noticeable improvement in microhardness, and a decrease in the early adhesion of *C. albicans* on the surface.	[114]
Helium	32.0 kHz, 13.0 kV, 2000 sccm	In vitro experiments found that a 5-minute CAP treatment significantly reduced the number of viable *C. albicans* cells and maintained the high cell viability of Vero cells (86.33 ± 10.45%), and it did not show delayed effects within 48 hours. In animal studies, a 5-minute CAP treatment significantly reduced the number of mycelia invading the tongue epithelium and significantly reduced the incidence of inflammation compared to nystatin.	[115]
Helium	32.0 kHz, 13.0 kV, 1 W, 2000 sccm	For 24 and 48 h *C. albicans* biofilms, 5 min of CAP treatment was significantly more effective than antifungal drugs (nystatin and amphotericin B). In addition, co-treatment of CAP with antifungal drugs did not enhance its antimicrobial capacity.	[116]
Argon	4300 sccm	Clinical studies have found a significant reduction in erythema after the combination of CAP with nystatin, and CHX compared to placebo during 2–6 weeks of antifungal therapy. There was no statistically significant difference in visual analog score values and frequency of moderate to severe *C. albicans* growth between the two groups after treatment.	[117]
Argon or Argon/Oxygen (100:1)	40 kHz, 10 kV, Argon: 50 sccm, Oxygen: 0.5 sccm	The ability of CAP to kill *C. albicans* was diminished by the addition of 1% oxygen. For 2-day *C. albicans* biofilms, Ar-CAP treatment was significantly better than 0.1% CHX, while the synergistic effect of the combination of them was not obvious. For *C. albicans* biofilms of 7 days or longer, Ar-CAP treatment alone was not effective and only showed significant antifungal effects when combined with CHX or NaOCl.	[118]
Nitrogen	1 kHz, 15.5 kV, 0.01μs, 45 ± 4 mJ	The medium treated with DBD-CAP showed significant inhibitory effects on both HSV-1-infected corneal epithelial cells and isolated human corneas, and it was biologically safe.	[119]
Argon	5 L/min, 2.5 atm	CAP treatment did not inhibit GFP expression in the HSV-1 genome, and it is hypothesized that CAP jets may only be effective at low viral doses.	[120]

Abbreviation: CAP, cold atmospheric plasma; CHX, chlorhexidine; sccm, standard cubic centimeter per minute; CFU, colony forming units; DBD, dielectric barrier discharge; NaOCl, sodium hypochlorite; EDTA, ethylenediaminetetraacetic acid; TSC, trisodium citrate; PDT, photodynamic therapy; SEM, scanning electron microscope; HSV: herpes simplex virus.

### 4.1. Enterococcus faecalis

Dental hard tissue defects, such as caries, can lead to acute or chronic pulpitis, pulp necrosis, and inflammation of periapical tissues. In such cases, perfect root canal treatment is necessary. However, persistently infected root canal treatment is a major clinical problem. *E. faecalis* is the primary pathogen for secondary apical periodontitis [121]. The anatomical structure of the root canal system is complex, and residual *E. faecalis* in lateral accessory canals is difficult to remove through conventional conditions and is resistant to commonly used antiseptics in endodontic treatment, such as calcium hydroxide. At the same time, other drugs like formaldehyde cresol and camphor have toxic side effects and are no longer used [122]. To combat *E. faecalis* biofilm and treat recalcitrant root canal infections, scholars have been looking for a new solution, which is where CAP comes in.

Several articles have demonstrated the effectiveness of CAP in killing *E. faecalis*. In a study by Theinkom et al. agar plates of *E. faecalis* were treated with ambient air CAP for 1–10 min, resulting in a 7–9 log reduction in CFU [97]. The treatment of a 24-hour *E. faecalis* biofilm for 5 min resulted in a 3-log reduction in CFU and a 5-log reduction after 10 min. This effect was comparable to 0.2% CHX, indicating that CAP is effective against *E. faecalis* in both planktonic and biofilm states. Cao et al. confirmed the inhibitory effect of CAP jet on *E. faecalis* and its biofilm [98]. Additionally, a 2-minute DBD-type CAP treatment reduced *E. faecalis* by 90%, indicating that CAP can achieve effective sterilization in a short period [99].

The impact of CAP treatment on removing *E. faecalis* biofilm from root canals was significant. In a study conducted by Pan et al. an argon/oxygen CAP jet was used to treat root canals that were infected with *E. faecalis* for 2–10 min [100]. The study found a significant reduction in CFU that was associated with the duration of the CAP treatment. Moreover, CAP treatment for 8–10 min was observed to be more effective in combating microorganisms than calcium hydroxide. Apart from the duration of treatment, the location in the root canal also affects the killing effect of CAP on *E. faecalis*. Kaya et al. conducted a study on the antimicrobial effect of CAP on *E. faecalis* in the root canal wall and dentin tubules [101]. The study found that helium/oxygen CAP was more effective in the middle 1/3 of the root canal than in the positive control sodium hypochlorite group (*p* < 0.05), while there was no significant difference in the crown and apical 1/3. Herbst et al. analyzed the bactericidal effect of CAP on infected dentin at different depths from 0 to 800μm [102]. The study found that the combined application of CAP with 2% CHX gave the best results overall, consistent with the conclusions drawn for the surface layer of 0–300μm. The difference was insignificant in the depth of 300–500 μm, while in the deeper layers of 500–800 μm, the combined treatment was surprisingly less effective than the application of CHX alone. This may be due to anatomical differences, such as sclerosis of dentin tubules reducing the depth of fluid penetration. However, this is only the author’s conjecture, and further experiments are needed to confirm the mechanism behind it.

In experiments conducted by Herbst, it was discovered that combining CAP with other treatments can significantly increase its effectiveness in killing *E. faecalis*. Huefner et al. found that CAP treatment alone was less effective in killing *E. faecalis* biofilms in isolated dental root canals than 3% NaOCl [103]. However, when CAP containing 1% oxygen was applied for a prolonged period, it significantly reduced the density of *E. faecalis*, and its combined treatment with NaOCl showed the best disinfecting effect. Du et al. tested the antibacterial properties of CHX-modified CAP jet on *E. faecalis* biofilms *in vitro*, in which the gas was first passed through 2% CHX, and then the low-temperature plasma jet was generated by a plasma generator [104]. They found that both CHX and CAP treatments caused a significant increase in dead bacteria in *E. faecalis* biofilms and multispecies biofilms on dentin discs, with both having comparable effects. A more significant bactericidal effect was demonstrated when the CHX-modified CAP jet was used. This suggests that pretreatment of the working gas may contribute to the efficient antimicrobial properties of the plasma. Similarly, Zhou et al. treated *E. faecalis* biofilms in root canals of extracted anterior teeth and found that the antimicrobial effect of 3% H_2_O_2_-modified helium CAP jets under the same conditions was significantly better than that of CAP treatment alone [105]. Tschang et al. also demonstrated that the combined therapy of EDTA or TSC with CAP had a synergistic effect on the killing of *E. faecalis* biofilm in both groups [106].

Photodynamic therapy (PDT) is a minimally invasive treatment that utilizes photosensitizers to produce ROS when exposed to light. This process is used to eliminate cancer cells. Compared to chemotherapy and radiotherapy, PDT has fewer side effects and lower systemic toxicity. It has been employed in treating various solid tumors [123]. In recent years, there has been growing interest in studying the antimicrobial effects of PDT, especially in its potential application in root canal therapy [124]. A study was conducted by Armand et al. [107] to evaluate the antimicrobial efficacy of CAP jet and PDT in root canals infected with *E. faecalis*. The study found that the bacteria were significantly reduced in all treatment groups. Still, the effect of the helium CAP jet containing a certain percentage of oxygen was the most significant due to the higher content of reactive oxygen species produced. Another study by Ballout et al. compared the effects of two different CAP sources (CAP jet and DBD-CAP), PDT and sodium hypochlorite, on biofilms in infected root canals [108]. The study found that sodium hypochlorite had the most significant effect. However, on the crown side of the root canal, the impact of PDT and argon CAP jet were comparable, and both had significantly lower colony-forming units than the DBD-CAP. In conclusion, the effect of CAP is affected by different plasma sources and working gases and is comparable to or possibly superior to PDT.

Li et al. conducted a study to evaluate the effectiveness of CAP jet in removing *E. faecalis* biofilm from dentin and to assess its mechanical safety [109]. The findings revealed that CAP treatment was highly effective in eradicating *E. faecalis* biofilm from root canals that were cultured for three weeks, and the efficacy increased with treatment time. For instance, a 12-minute CAP treatment completely eliminated *E. faecalis* biofilm, whereas calcium hydroxide paste and 2% CHX gluconate gel treatments for 7 days did not. Furthermore, CAP-treated root canal dentin showed no significant changes in microhardness or roughness, indicating that the treatment was mechanically safe. Yao et al. further investigated the efficacy of CAP *in vivo* by establishing an experimental periapical inflammation model in Beagle dogs [110]. The results showed a significant increase in BV/TV and a more apparent reduction in the volume of periapical lesions in the CAP-treated group compared to the group treated with 2% CHX. Histologic staining revealed fewer inflammatory cells and bacterial residues in CAP-treated apical tissues, indicating good antibacterial efficacy *in vivo*.

In conclusion, it has been found that CAP has a good ability to counteract the effects of *E. faecalis*, both *in vitro* as a planktonic form or in the form of biofilm and *in vivo* within the root canal system. Its effectiveness is time-dependent and can be further enhanced through synergistic means. Moreover, the treatment is mechanically safe, providing a viable option for the clinical treatment of endodontic and periapical diseases. Compared to the recent PDT treatment for root canal therapy, CAP is equally effective. However, most experiments are still limited to *in vitro* studies of plasma, and there are relatively few studies on recalcitrant root canal infections *in vivo*. Furthermore, it is still unclear whether the root canal treatment process using CAP meets the requirements of organismal biosafety, which requires further improvement by scholars.

### 4.2. Candida albicans

*C. albicans* is a type of fungus commonly found in most people’s mouths. Usually, this fungus does not cause any harm to healthy individuals, but people with weakened immune systems are at risk of developing infections known as “oral candidiasis” [125]. People with HIV are particularly susceptible to *C. albicans* infections, and wearing removable dentures for long periods can also increase the likelihood of developing oral candidiasis [126]. *C. albicans* can adhere to surfaces, invade tissues, and adapt to changes in its environment in the mouth due to its ability to produce adhesins and invasins, secrete enzymes, and change from yeast to a mycelial form [127]. Additionally, *C. albicans* has been associated with oral squamous carcinoma [128]. Antifungal agents such as nystatin are the most widely used treatment [129]. However, due to increased fungal resistance, researchers are exploring alternative treatments, such as CAP therapy, to combat this issue [130].

CAP contains various active ingredients that may inhibit the growth of *C. albicans. In vitro* experiments by Rupf and Kim et al. [51,111] have confirmed that CAP jets have a time-dependent killing effect on *C. albicans*. Pu et al. found that the CAP jet with air as its working gas can significantly disrupt *C. albicans* biofilm, with a 90% kill rate after 20 s of treatment and no viable fungus detected after 55 s [112]. The effectiveness of CAP jets in inhibiting fungal growth can be affected by different flow rates and treatment distances. In a study by Nishime et al. helium CAP jets with flow rates of 2.0 SLM and 4.0 SLM were projected onto agar plates [113]. Although the increasing gas flow rate did not significantly change the area of the *C. albicans* inhibition zone, it disrupted the shape and homogeneity of the zone. At higher flow rates, the edges of the inhibition zone became irregular, which could be attributed to hydrodynamic effects such as turbulent mixing and buoyancy. Furthermore, increasing the distance between the CAP jet and the agar plate weakened the inhibition zone of *C. albicans*, but not that of *E. faecalis*. Therefore, the distance between the CAP jet and the target is essential in determining its effectiveness in inhibiting fungal growth. In addition, Pan et al. found that the argon/oxygen jet could modify the surface of acrylic resin to improve the hydrophilicity of the material, reduce the early adhesion of *C. albicans*, and enhance the retention capacity [114].

Borges et al. conducted animal experiments based on their *in vitro* experiments [115]. They initially tested *C. albicans* biofilms and Vero cells *in vitro* using helium CAP jets to determine the plasma parameter with low cytotoxicity and anti-biofilm effect. After that, they treated mice with oral candidiasis with this parameter, and healthy mice were treated to verify its *in vivo* compatibility. Histological analysis showed a significant reduction in inflammation and tissue invasion after CAP treatment, while no histological changes were observed in healthy mice. This suggests that the jet has anti-inflammatory and anti-*Candida* efficacy along with biosafety, opening new possibilities for the clinical treatment of oral candidiasis.

Several studies have examined the effectiveness of CAP in combination with other antifungal drugs. Leite et al. found that CAP was more effective against *C. albicans* biofilms than conventional antifungal agents. Still, they found no synergistic effects of CAP with nystatin or amphotericin B [116]. Preissner et al. conducted a study on treating odontogenic stomatitis using a combination of CAP jet, antifungal agents, and CHX [117]. According to the results, there was a significant reduction in the area of erythema in the experimental group compared to the control group after two weeks, with an extremely high correlation observed at weeks four and five. However, there was no significant difference in the frequency of *C. albicans* colony-forming units before and after treatment, except for moderate to severe growth on the test side. This suggests that the combination treatment had a more rapid and extensive effect on erythema remission but had no significant impact on *C. albicans*. Matthes et al. conducted a study on the effectiveness of argon CAP, CHX, and NaOCl on *C. albicans* biofilms of varying maturity levels [118]. The study found that argon CAP alone was more effective than CHX for 2-day-old biofilms, but combining the two did not have a significant synergistic effect. However, for biofilms of 7 days or older, argon CAP alone was not effective and only showed substantial effects when combined with CHX or NaOCl. The research suggests that the maturity of *C. albicans* biofilm is an essential factor in determining the therapeutic effect of the treatment. Therefore, treatment protocols should be adjusted for biofilms of different maturity levels, and mechanical pretreatment is necessary when utilizing plasma to treat long-term *C. albicans* biofilms.

Based on this, CAP is a promising treatment for diseases such as denture stomatitis caused by *C. albicans*. The combination treatment strategy by plasma can help characterize the regression of denture stomatitis. However, it is essential to note that the combination treatment may not be significant in some cases against *C. albicans*. This may be due to differences in test results, biofilm maturity, or the number of patients. Therefore, further relevant studies are needed to explore the exact effect of combination therapy. Finally, CAP has crucial clinical value as it decontaminates the *C. albicans* biofilm on the denture base surface and modifies it to improve hydrophilicity, inhibit *C. albicans* adhesion, and improve retention. This is beneficial for the restoration of removable partial dentures and complete dentures.

### 4.3. Herpes simplex virus

Herpes simplex virus (HSV) is a type of DNA virus that primarily causes infections in the oral and maxillofacial areas. However, it can also lead to other conditions, such as encephalitis, conjunctivitis, and neonatal herpes [131]. HSV-1 typically begins its infection by replicating in the nucleus and producing characteristic vesicles after invading the epithelium of the oral mucosa. Then, it moves along sensory nerves to the trigeminal ganglion, which integrates with the host’s DNA and enters a latent stage. The exact process by which the virus is reactivated from this stage is still not fully understood. Once it reactivates, the virus spreads back from the ganglion and causes new skin and mucosal lesions [132]. Mohamed et al. [133] pointed out that CAP can potentially prevent viral infections and treat related diseases.

Filipić et al. demonstrated in their review that cold plasma has a good inactivation effect on a wide range of viruses, whereas not many studies have been done on herpes simplex virus [134]. Alekseev et al. discovered that the medium treated with DBD-CAP could inhibit the lesioning effect of HSV-1 in human corneal epithelial cells, reduce viral replication, and limit the expansion of HSV-1 plaques [119]. Bunz et al. evaluated the transduction efficiency of HSV-1 in Vero and SH-SY5Y cells after argon CAP jet treatment by measuring the expression level of a reporter gene GFP [120]. In contrast to Alekseev’s findings, it was observed that the jet treatment did not inhibit GFP expression in the HSV-1 genome, and it was postulated that an excess virus may disrupt the CAP effect. Therefore, the number of viruses was controlled in the subsequent experiments. When the viral load was decreased 100-fold, a slight but detectable decrease in GFP expression levels was observed. Additionally, a significant reduction in HSV-1 genomic expression was observed at 3 h of viral infection after CAP treatment, while the expression at 24 h was insignificant, which could be a result of viral replication. To some extent, these findings confirm the speculation mentioned above that the argon CAP jet has an antiviral effect at low viral doses.

In conclusion, it appears that DBD-type plasma is potentially more effective than the jet in combating viruses. However, the effectiveness may vary based on factors such as the type of working gas, the cells used, etc. There isn’t enough experimental evidence to confirm this. Currently, most research on CAP’s antimicrobial effects is focused on bacteria and fungi. Studies on viruses are inadequate, and further research is needed to understand the antiviral mechanism and effects of CAP.

## 5. CAP against biofilms

The oral cavity contains a large and diverse population of microorganisms, usually in the form of multispecies biofilms attached to various surfaces. These biofilms are composed of microbial structures enclosed in an extracellular polymeric matrix [135]. In this structure, microorganisms are tightly bound together by extracellular substances, which enable them to resist environmental disturbances and contribute to oral health [136]. However, changes in the oral environment can compromise these microbial interactions, leading to oral infectious diseases such as dental caries, periodontal disease, and oral mucosal diseases [35]. To be therapeutically effective, biofilm activity must be removed or inhibited. Unfortunately, the tight binding of various microorganisms in biofilms makes them resistant to antibiotics [137,138]. Therefore, there is an urgent need to find a new approach to combat oral biofilms that can replace antibiotics. Rao et al. stated that CAP is a notable strategy to eliminate and prevent mixed biofilms [139]. Table 5 shows some relevant studies on CAP against biofilms.

**Table 5.** The effect of CAP against biofilms.

CAP carrier gas	CAP parameters	Results	References
Argon	8 W, 5000 sccm	The 30-second CAP treatment significantly reduced the variation of *S. mutans*, *S. gordonii*, and *S. sanguinis* formed single or multi-species biofilm viability.	[140]
Helium/ Oxygen (99:1)	8 kV, 8 kHz, 1.6 μs, 1 L/min	A 5-minute CAP treatment can penetrate a 10-day-old *P. gingivalis* biofilm with a thickness of about 15μm, which is equivalent to the 30 layers of bacteria, effectively inactivating all of them. However, the structure of the majority of the dead cells will not be destroyed.	[141]
Argon	8 mA, 3 kV, 10 W	Bacteria in a 20µm thick biofilm of *S. mutans*, grown for 3 days, were inactivated by over 90% after 1 minute of CAP treatment and reached over 95% after 5 minutes.	[48]
Air	20 kHz, 6 mA, 100000μs, 12V, 60 mW	The 5-minute CAP treatment not only inactivated the bacteria in the top layer of the 25.5μm thick *E. faecalis* biofilm but also penetrated the bottom layer to kill the bacteria.	[142]
Argon or Argon/Oxygen	40 kHz, 10 kV, Argon: 50 sccm, Oxygen: 0.5 sccm	For 2-day *C. albicans* biofilms, CAP treatment was significantly more effective than 0.1% CHX. For biofilms of 7 days or more, CAP alone was not effective and only showed significant antifungal effects when combined with CHX or NaOCl.	[118]
Air	A:20 kHz, 13V, 0.03 mW/cm^2^ B:30 kHz, 8V, 500 sccm, 0.18 mW/cm^2^	The two CAPs had a similar antimicrobial effect on *Pseudomonas aeruginosa* and *Staphylococcus epidermidis* biofilms as 0.1% CHX. However, the antimicrobial effect of CAP might be limited by the formation of bacterial cell debris on the biofilm surface.	[143]
Argon	3100 sccm, 1.82 MHz	After a 10-minute CAP treatment, there was a 3.5 log reduction in colony-forming units for three Gram-negative bacteria, compared with only a 0.5–2 log reduction for Gram-positive bacteria. Additionally, CAP treatment for 1–3 minutes resulted in a greater decrease in *Enterobacter cloacae* with thicker cell walls than in *Pseudomonas aeruginosa.*	[83]
Argon	13.56 MHz, 0.15W/0.9W/1.6W, 500 or 1000 sccm	Gram-negative bacteria were more susceptible to CAP treatment. In addition, power and treatment time were also closely involved in the antimicrobial effect.	[144]
Helium/ Oxygen (1000:1)	2 L/min, 10 Hz, 50000μs	CAP demonstrated an immediate killing effect on *E. faecalis* biofilm similar to CHX. Additionally, it exhibited a sustained killing effect, similar to CHX, for up to one week.	[145]
Argon	10 W, 10 L/min	CAP treatment increased the surface energy of dental zirconia and inhibited *S. mutans* adhesion for at least 48 hours.	[18]
Argon/Oxygen	3 W, 6 mA, 0.5 kV, Argon: 3000 sccm, Oxygen: 30 sccm	CAP treatment not only instantly inactivated biofilms but also inhibited their growth after treatment.	[146]
Ambient air	50 kHz, 8 W	The 30-second treatment completely killed *F. nucleatum* and *P. intermedia*, and *T. forsythia* was completely killed within 60 seconds. CAP treatment of dentin specimens did not significantly reduce the amount of biomass covering the bacterial and biofilm matrices, but the metabolic activity was reduced by approximately 70% after 60 seconds of treatment and by approximately 95% after 120 seconds.	[74]
Argon/Oxygen (98:2)	18 kV, 10 kHz, 5 L/min	After 10 minutes of CAP treatment, mycelia and bacteria spheres within the *E. faecalis* biofilm disappeared from the surface of dentin tubules, while fragments of the biofilm remained on the surface of the tubes with no significant change in thickness.	[100]
Air	80 kV, 50 Hz	The thickness of *Pseudomonas aeruginosa* biofilms did not change after CAP treatment. However, the toxicity of the biofilm was significantly reduced due to the decrease in the expression of quorum sensing-regulated virulence factors.	[147]
Helium/ Oxygen (200:1)	6 kV, 20 kHz	CAP attenuated biofilm toxicity by reducing the ability of acyl-homoserine lactones to depend on quorum sensing.	[148]
Air	135 W, 60 Hz, 18 m/s	CAP treatment removed microorganisms from mature biofilms that conventional antimicrobials usually fail to eradicate. This may be because CAP destroyed polysaccharides in the biofilm matrix.	[45]

Abbreviation: CAP, cold atmospheric plasma; sccm, standard cubic centimeter per minute; CHX, chlorhexidine; NaOCl, sodium hypochlorite.

Figueira and colleagues have discovered that CAP can prevent biofilms from forming by one or more streptococci [140]. However, CAP has limitations when it comes to penetrating thick biofilms. Therefore, it is crucial to ensure that it permeates the entire biofilm [149]. Currently, the effectiveness of CAP in penetrating biofilms of different thicknesses is not well studied and may be affected by the type of biofilm and gas used. For example, Xiong et al. found that the helium/oxygen CAP jet could penetrate *P. gingivalis* biofilm with a thickness of 15 μm [141]. Meanwhile, Hong et al. found that when argon was used as the working gas, the plasma material could penetrate the *S. mutans* biofilm with a thickness of 20 μm, which caused severe cellular damage to the top surface of the biofilm [48]. Similarly, Pei et al. used an air plasma jet to penetrate *E. faecalis* biofilm with a thickness of 25.5 μm [142]. However, more research is needed to determine the penetration ability of CAP.

In addition, it has been observed that the effectiveness of CAP on biofilms depends on their maturity. CAP was found to be more effective against early biofilms than long-term biofilms. The experiments by Matthes et al. showed that the inhibitory effect of argon CAP on mature *C. albicans* biofilms was significantly reduced [118]. This was explained in another paper, which suggested that the top of the biofilm may have formed a debris layer after CAP action, inhibiting the deeper plasma effects on the biofilm [143]. The debris layer is formed due to coagulation of the biofilm matrix and cytoplasm after cell wall disruption by plasma products. This explains why Gram-negative bacteria are more susceptible to CAP than positive strains. Mai-Prochnow et al. pointed out that there was a correlation between CAP inactivation in bacteria and the thickness of the cell wall, with biofilms of Gram-negative bacteria with thinner cell walls being inactivated more rapidly than biofilms of Gram-positive bacteria with thicker cell walls [83], and Puac et al. came to the same conclusion [144]. This suggests that mechanical pretreatment must first be performed when applying CAP to mature biofilms, especially those with thick cell walls.

In addition to its immediate killing effect, CAP can also have a sustained impact on biofilms. Tang et al. showed that CAP had a sustained killing effect on *E. faecalis* biofilms similar to that of CHX, which lasted at least a week [145]. Park et al. found that CAP treatment increased the surface energy of dental zirconia and inhibited *S. mutans* adhesion for at least 48 h [18]. Further research is needed to determine how long this window lasts and whether CAP parameters and biofilm type influence it. Hong et al. re-cultured the CAP-treated *S. mutans* biofilm and found that the growth of the biofilm was inhibited [146]. The newly formed *S. mutans* biofilm showed increased metabolic activity and decreased resistance to antibiotics and tolerance to oxidative stress, suggesting that CAP treatment is beneficial for the long-term control of the biofilm.

Various studies have investigated the effectiveness of CAP against biofilms and the underlying mechanisms. Jungbauer et al. found that CAP treatment reduced biofilms’ viability, but the biofilm base’s quality was unaffected [74]. Pan et al. also confirmed that after CAP treatment, mycelia and bacteria spheres within the *E. faecalis* biofilm disappeared from the surface of dentin tubules, while fragments of the biofilm remained on the surface of the tubes with no significant change in thickness [100]. Ziuzina et al. discovered that although the thickness of *Pseudomonas aeruginosa* biofilms did not change after CAP treatment, the toxicity of the biofilm was significantly reduced. This was due to a decrease in the expression of quorum sensing-regulated virulence factors [147]. Flynn et al. reported that CAP attenuated biofilm toxicity by reducing the ability of acyl-homoserine lactones to depend on quorum sensing [148]. Duarte et al. found that CAP treatment removed microorganisms from mature biofilms that conventional antimicrobials usually do not eradicate. This was possibly due to the disruption of polysaccharides in the biofilm matrix, which exposed the microorganisms, affecting the amount of bacteria in the biofilm and their ability to survive [45]. Therefore, CAP could be a helpful method for disinfecting biofilms.

In conclusion, CAP treatment helps to reduce biofilm activity and inhibit biofilm formation. It’s important to consider that the thickness of the biofilm, its maturity, and the types of microorganisms present can impact the effectiveness of CAP in combating biofilms. Further investigation into the anti-biofilm effect of CAP is needed to understand the treatment outcomes better. Additionally, applying CAP in a more personalized way by fully considering the various influencing factors in future clinical treatments is essential.

## 6. Principles of CAP’s anti-microbial action

**Figure 2.**
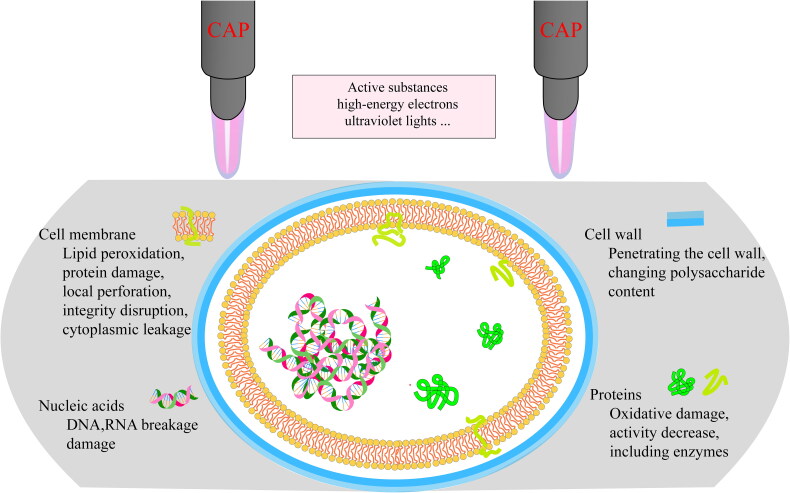
The mechanism of action of CAP on pathogenic microorganisms.

CAP is a type of plasma generated at atmospheric pressure that can produce some inhibitory effects on bacteria, fungi, and viruses. The mechanism of action of CAP on pathogenic microorganisms is summarized in Figure 2.

How does CAP work to inhibit microorganisms? The effect of CAP on fungal cell walls has been studied, and it was found that after 120 s of helium CAP treatment, the structure of the cell walls changed significantly: β-glucans decreased, chitin increased, and permeability increased [150]. Changes in protein content were also observed. Another study looked at the relationship between the antimicrobial effect of CAP and the thickness of bacterial cell walls [83]. It was found that after 10 min of CAP treatment, the colony-forming units of three Gram-negative bacteria (*Pseudomonas libanensis*, *Pseudomonas aeruginosa*, *Enterobacter cloaceae*) with thinner cell walls decreased by up to 3.5 logs, while Gram-positive bacteria (*Staphylococcus epidermidis*, *Kocuria carniphila*, and *Bacillus subtilis*) decreased by only 0.5–2 log. This suggests that the thickness of the cell wall is an important factor in limiting the antimicrobial effect of CAP. Additionally, the study found that colony-forming units of *Enterobacter cloaceae*, which have thicker cell walls than *Pseudomonas aeruginosa*, declined more when treated with CAP for 1–3 min. This implies that CAP has other targets besides the cell wall.

Cell membranes are important targets for CAP action [151–153]. Soler-Arango et al. utilized Fourier Transform infrared spectroscopy to demonstrate that CAP causes structural damage to bacterial cells by inducing lipid peroxidation of cell membranes time-dependently [154]. Similar conclusions were reached by Joshi et al. [155]. Zhang et al. analyzed the antibacterial effect of plasma in terms of the etching of bacterial cell walls and membranes, and Kobzev et al. showed that CAP reduces the strength of the bacterial cell wall and causes damage to the cell membrane [156,157].

CAP can also cause harm to other biomolecules. The SOS response is closely related to DNA damage [158]. Sharma et al. discovered that the expression of SOS response regulators was heavily upregulated in *E. coli* treated with argon CAP, which corroborated the presence of DNA damage [159]. Additionally, they observed differential expression of several oxidative stress response genes, including OxyS, confirming the oxidative stress effect of CAP. Ebrahimi-Shaghaghi et al. also demonstrated that CAP treatment inhibits the expression of *CaMCA1* and *HSP90* genes in *C. albicans*, thereby reducing the pathogenicity of the fungus and inducing death of *C. albicans* [160,161]. Barkhade et al. confirmed the alteration of the structure of bacterial proteins after CAP treatment by circular dichroism [162], and Winter et al. similarly demonstrated that plasma can target proteins [163]. Guo et al. demonstrated that CAP, especially monomorphic oxygen, can cause the degradation of proteins and nucleic acids in phages, which explains the principle of antiviral action [164]. Therefore, combating viral infection by CAP is expected to be a novel strategy.

So, what plays a key role in CAP? Graves noted that the cellular effects of CAP appear to mainly involve, either directly or indirectly, the RONS created by CAP in air environments [165]. Laroussi et al. have long demonstrated that RONS play the most critical role in inhibiting microorganisms, with UV radiation and other factors playing a secondary role [166]. Deng et al. reached similar conclusions [167]. Specifically, RONS cause physical damage to cell membranes, whereas UV radiation does not. However, both the RONS and UV radiation produced by CAP can modify DNA bases, induce DNA backbone breaks, and cause protein inactivation and oxidative stress. In addition, the unseparated effluent was found to be more effective in removing bacteria than exposing them to only plasma-released reactive species. This suggests that active species are CAP’s primary, but not the only, bactericidal factor [168]. Thus, the combined effect of factors such as RONS and UV radiation is the reason for CAP’s antimicrobial capability, with RONS playing the primary role [168–170].

There are many types of RONS, such as H_2_O_2_, O_2_^−^, OH^−^ NO, O, NO_3_^−^, NO_2_^−^, and ONOO; who plays a more critical role? Murakami et al. found that the reactive neutral species, atomic oxygen, singlet delta oxygen, and ozone dominated the substances produced by CAP [171]. Puac et al. explored the effects of CAP on bacterial suspensions [144]. They found that CAP treatment produced H, OH, and NO radicals. Among these, NO was identified as the most critical radical, demonstrating strong antimicrobial effects against Gram-negative and Gram-positive bacteria. It is important to note that CAP-generated RONS work together as a complex. When fluids were artificially supplemented with the same concentrations of H_2_O_2_ and NO_2_^−^, they did not produce the same effects as CAP. This suggests that the entire RONS complex is essential for CAP to exert its biological effects [172].

In conclusion, the biological effects of CAP arise from the synergistic action of various factors, primarily RONS. These factors ultimately damage multiple components of microorganisms, including cell membranes, proteins, and nucleic acids, leading to cell death. To effectively apply CAP in clinical settings, it is crucial to investigate its effects on normal cells further and thoroughly analyze the differences between CAP and pathogenic microorganisms. This ensures the safety and reliability of its application.

## 7. Conclusions and future perspectives

Microorganisms in the oral cavity typically exist in a delicate balance. When this balance is disrupted, it can lead to a range of oral health issues, including dental caries, periodontal disease, endodontic conditions, and mucosal diseases. CAP, which utilizes RONS as its core mechanism, can effectively penetrate the cell walls of pathogenic bacteria. This process damages bacterial cell membranes by targeting various biomolecules, such as lipids, proteins, and nucleic acids. As a result, CAP effectively disrupts bacterial biofilms, reduces pathogenicity, and serves a therapeutic purpose. Moreover, studies suggest that repeated use of CAP does not result in increased bacterial resistance, unlike conventional antibiotic therapies. Therefore, CAP, as a non-invasive treatment that is also effective against fungi and viruses, offers a promising new strategy for addressing infectious diseases in the oral cavity [173,174].

There is still a long way to go before CAP can be used in clinical settings. CAP is a source of ROS, and we must ensure it does not cause oxidative stress in tissues. ROS can be both beneficial and harmful depending on the dose [175]. They can promote tissue repair at low doses, while excessive amounts can cause cell death. However, we are uncertain about what constitutes a safe dose of CAP and how to measure it [176]. The duration and power of CAP treatment do not always determine the dose; different types of CAP might have different effects under the same conditions [177,178]. Therefore, researchers propose using the concept of equivalent total oxidation potential (ETOP) to determine the dose [179]. We need to better understand the dosage that balances clinical effectiveness and biosafety of CAP treatment. Some studies have been conducted to determine the effects of different doses of CAP on cells and tissues. For instance, research on gingival epithelium showed that CAP treatment for 1–3 min was not toxic and had high cell viability, with 3-minute treatment being slightly more cytotoxic than 1-minute treatment [69]. Similarly, another study found that a 3-minute CAP treatment maintained high cell viability of Vero cells without any delayed effects. A slight decrease in cell viability was observed after a 5-minute treatment, but it was still above 70%, indicating a lower cytotoxicity of CAP at this dose. Using this dose, researchers treated mice with *C. albicans* and found that a 5-minute CAP treatment significantly reduced tissue invasion by the fungus without causing significant harm to healthy tissue. This suggests that a 5-minute treatment with CAP is biologically safe and effective in treating *C. albicans* infection [115].

It is crucial to assess the impact of CAP based on various factors, with one of the most significant being the type and parameters of CAP. A study found that for *C. albicans* biofilms on titanium disks, CAP jets were not as effective as 0.1% CHX, whereas the results for hollow DBD and volume DBD were significantly better than those for CHX [180]. In contrast, another study found that in the coronal portion of the root canal, CAP jets killed *E. faecalis* better than DBD [108]. The differences in these two sets of studies may be attributed to variations in ETOP stemming from differences in CAP parameters such as power. Therefore, individualized parameters for different CAP devices must be set to obtain the exact effect. Another important factor is the type of CAP carrier gas used. Helium and argon are the most commonly used working gases. Adding a small amount of oxygen to the gas to generate more ROS is also common. A study found that a CAP jet with 1% oxygen added was more bactericidal to *P. gingivalis* biofilms than argon alone [72]. In contrast, another study found that argon CAP alone was significantly more effective in killing *C. albicans* than mixed with 1% oxygen, which was more pronounced after 5–10 min of treatment. This difference can be attributed to the quenching of argon-excited substances.

When using CAP for treatment, the type of targeted bacteria plays a crucial role in the treatment’s effectiveness. Gram-positive bacteria have thicker cell walls (20–80 nm) compared to Gram-negative bacteria (<10 nm), so the CAP treatment has a more substantial effect on Gram-negative bacteria [83]. Additionally, the duration of the CAP treatment and the distance from the sample also impact its effectiveness. Although some studies have concluded that the distance does not affect the bactericidal effect of CAP [71], others have found that the appropriate distance provides the temporal and spatial conditions for the production of the active substances, making CAP more effective [53]. Shortening the treatment distance may help to kill *C. albicans*, but the effect on *E. faecalis* and *P. aeruginosa* is not constant, and the best antimicrobial effect is shown by different treatment distances at different treatment times [113]. Last but not least, it is important to consider the environment in which the CAP is used. When the surrounding air has high humidity, the density of ROS produced by CAP decreases, leading to an overall decrease in plasma reactivity. However, in such conditions, the diversity of substances produced by CAP increases. This includes an increase in nitrogen and hydrogen radicals and the enhancement of negative ions such as O_3_^−^, NO_2_^−^, and NO_3_^−^. Additionally, the electronegativity increases [171].

It is clear that various factors influence the biological effects of CAP. Therefore, it is essential to establish a comprehensive evaluation system to assess the impact of CAP. More detailed studies should be conducted for different types of CAP devices, focusing on aspects such as CAP’s antimicrobial properties, anti-inflammatory effects, biosafety performance, and additional relevant experiments both *in vitro* and *in vivo*. The more extensive and in-depth the research, the sooner we can realize the clinical applications of CAP. Additionally, it is important to remember that the oral cavity is a complex environment that contains numerous microorganisms. When combined with the host’s immune response, the results from *in vitro* experiments may not necessarily align with those from *in vivo* studies. Consequently, a significant number of *in vivo* experiments are crucial, as better results in these studies will enhance the potential for future clinical applications. This is a vital consideration for future CAP research in dentistry.

## Data Availability

Data sharing is not applicable to this article as no new data were created or analyzed in this study.

## References

[1] Reuter S, Von Woedtke T, Weltmann K-D. The kINPen—A review on physics and chemistry of the atmospheric pressure plasma jet and its applications. J Phys D: Appl Phys. 2018;51(23):233001. doi: 10.1088/1361-6463/aab3ad.

[2] Braný D, Dvorská D, Halašová E, et al. Cold atmospheric plasma: a powerful tool for modern medicine. Int J Mol Sci. 2020;21(8):2932. doi: 10.3390/ijms21082932. 32331263 PMC7215620

[3] Lu X, Liu D, Xian Y, et al. Cold atmospheric-pressure air plasma jet: physics and opportunities. Phys Plasmas. 2021;28(10):100501. doi: 10.1063/5.0067478.

[4] Tendero C, Tixier C, Tristant P, et al. Atmospheric pressure plasmas: A review. Spectrochim Acta, Part B. 2006;61(1):2–30. doi: 10.1016/j.sab.2005.10.003.

[5] Duarte S, Panariello BHD. Comprehensive biomedical applications of low temperature plasmas. Arch Biochem Biophys. 2020;693:108560. doi: 10.1016/j.abb.2020.108560. 32857998 PMC7448743

[6] Gan L, Zhang S, Poorun D, et al. Medizinische Anwendungen von nicht-thermischem Atmosphärendruckplasma in der Dermatologie. J Dtsch Dermatol Ges. 2018;16(1):7–14. doi: 10.1111/ddg.13373_g. 29314681

[7] Laroussi M. Cold plasma in medicine and healthcare: the new frontier in low temperature plasma applications. Front Phys. 2020;8:528206. doi: 10.3389/fphy.2020.00074.

[8] Fadeeva IV, Kalita VI, Komlev DI, et al. In vitro properties of manganese-substituted tricalcium phosphate coatings for titanium biomedical implants deposited by arc plasma. Materials (Basel). 2020;13(19):4411. doi: 10.3390/ma13194411. 33022953 PMC7579245

[9] Liu Y-C, Lee Y-T, Huang T-C, et al. In vitro bioactivity and antibacterial activity of strontium-, magnesium-, and zinc-multidoped hydroxyapatite porous coatings applied via atmospheric plasma spraying. ACS Appl Bio Mater. 2021;4(3):2523–2533. doi: 10.1021/acsabm.0c01535. 35014370

[10] Afrasiabi M, Tahmasebi G, Eslami E, et al. Cold atmospheric plasma versus cisplatin against oral squamous cell carcinoma: a mitochondrial targeting study. Iran J Pharm Res. 2022;21(1):e124106. doi: 10.5812/ijpr-124106. 36942058 PMC10024331

[11] Saadawy H, Fathi EM, Elsayed I, et al. Treatment of ­hepatocellular carcinoma with in situ generated plasma-activated air-driven water mist. Plasma Processes & Polymers. 2023;20(9):e2200234. doi: 10.1002/ppap.202200234.

[12] Peng S, Qi M, Zhang H, et al. Discharge characteristics of a microsecond pulse power supply driven air plasma jet and its anticancer cell effect. Physics of Plasmas. 2022;29(1):013504. doi: 10.1063/5.0069851

[13] He R, Li Q, Shen W, et al. The efficacy and safety of cold atmospheric plasma as a novel therapy for diabetic wound in vitro and in vivo. Int Wound J. 2020;17(3):851–863. doi: 10.1111/iwj.13341. 32168435 PMC7949340

[14] Guo P, Liu Y, Li J, et al. A novel atmospheric-pressure air plasma jet for wound healing. Int Wound J. 2022;19(3):538–552. doi: 10.1111/iwj.13652. 34219379 PMC8874047

[15] Abbasi E, Mehrabadi JF, Nourani M, et al. Evaluation of cold atmospheric-pressure plasma against burn wound infections and gene silencing. Iran J Microbiol. 2021;13(4):544–552. doi: 10.18502/ijm.v13i4.6982. 34557284 PMC8421582

[16] Sakudo A, Toyokawa Y, Imanishi Y, et al. Crucial roles of reactive chemical species in modification of respiratory syncytial virus by nitrogen gas plasma. Mater Sci Eng C Mater Biol Appl. 2017;74:131–136. doi: 10.1016/j.msec.2017.02.007. 28254277

[17] Huang W-K, Weng C-C, Liao J-D, et al. Capillary-tube-based micro-plasma system for disinfecting dental biofilm. Int J Radiat Biol. 2013;89(5):364–370. doi: 10.3109/09553002.2013.756594. 23216281

[18] Park C, Park S-W, Yun K-D, et al. Effect of plasma treatment and its post process duration on shear bonding strength and antibacterial effect of dental zirconia. Materials (Basel). 2018;11(11):2233. doi: 10.3390/ma11112233. 30423984 PMC6266075

[19] Pan Y-H, Lin JCY, Chen MK, et al. Glow discharge plasma treatment on zirconia surface to enhance osteoblastic-like cell differentiation and antimicrobial effects. Materials (Basel). 2020;13(17):3771. doi: 10.3390/ma13173771. 32859067 PMC7503232

[20] Smeets R, Henningsen A, Heuberger R, et al. Influence of UV irradiation and cold atmospheric pressure plasma on zirconia surfaces: an in vitro study. Int J Oral Maxillofac Implants. 2019;34(2):329–336–336. doi: 10.11607/jomi.7017. 30521659

[21] Yang Y, Zheng M, Li J, et al. Inhibition of bacterial growth on zirconia abutment with a helium cold atmospheric plasma jet treatment. Clin Oral Investig. 2020;24(4):1465–1477. doi: 10.1007/s00784-019-03179-2. 31940064

[22] Ji M-K, Moon B-K, Kim H-S, et al. Assessment of inhibition of biofilm formation on TiO_2_ nanotubes according to non-thermal plasma treatment conditions and the elapsed time in the atmosphere. J Nanosci Nanotechnol. 2020;20(9):5742–5745. doi: 10.1166/jnn.2020.17658. 32331171

[23] Huang H-L, Tsai M-T, Lin Y-J, et al. Antibacterial and biological characteristics of tantalum oxide coated ­titanium pretreated by plasma electrolytic oxidation. Thin Solid Films. 2019;688:137268. doi: 10.1016/j.tsf.2019.04.043.

[24] Yue J, Jin Z, Poon HLE, et al. Osteogenic and antibacterial activity of a plasma-sprayed CeO_2_ coating on a Titanium (Ti)-based dental implant. Coatings. 2020;10(10):1007. doi: 10.3390/coatings10101007.

[25] Kim H-S, Yang S-Y, Choi EH, et al. Adhesion between epoxy resin-based fiber post and dental core resin ­improved by non-thermal atmospheric pressure plasma. Appl Sci. 2020;10(7):2535. doi: 10.3390/app10072535.

[26] Liu Y-C, Hsieh J-P, Chen Y-C, et al. Promoting porcelain–zirconia bonding using different atmospheric pressure gas plasmas. Dent Mater. 2018;34(8):1188–1198. doi: 10.1016/j.dental.2018.05.004. 29784462

[27] Dolci LS, Liguori A, Panzavolta S, et al. Non-equilibrium atmospheric pressure plasma as innovative method to crosslink and enhance mucoadhesion of econazole-loaded gelatin films for buccal drug delivery. Colloids Surf B Biointerfaces. 2018;163:73–82. doi: 10.1016/j.colsurfb.2017.12.030. 29278802

[28] Labay C, Hamouda I, Tampieri F, et al. Production of reactive species in alginate hydrogels for cold atmospheric plasma-based therapies. Sci Rep. 2019;9(1):16160. doi: 10.1038/s41598-019-52673-w. 31695110 PMC6834627

[29] ŽIvanić M, Espona-Noguera A, Verswyvel H, et al. Injectable plasma-treated alginate hydrogel for oxidative stress delivery to induce immunogenic cell death in osteosarcoma. Adv Funct Mater. 2024;34(14):2312005. doi: 10.1002/adfm.202312005.

[30] Egghe T, Morent R, Hoogenboom R, et al. Substrate-independent and widely applicable deposition of antibacterial coatings. Trends Biotechnol. 2023;41(1):63–76. doi: 10.1016/j.tibtech.2022.06.003. 35863949

[31] Buxadera-Palomero J, Canal C, Torrent-Camarero S, et al. Antifouling coatings for dental implants: polyethylene glycol-like coatings on titanium by plasma polymerization. Biointerphases. 2015;10(2):029505. doi: 10.1116/1.4913376. 25766480

[32] Rodriguez-Fernandez JC, Pastor F, Barrera Mora JM, et al. Bacteriostatic poly ethylene glycol plasma coatings for orthodontic titanium mini-implants. Materials (Basel). 2022;15(21):7487. doi: 10.3390/ma15217487. 36363077 PMC9654847

[33] Stasic JN, Pficer JK, Milicic B, et al. Effects of non-thermal atmospheric plasma on dentin wetting and adhesive bonding efficiency: systematic review and meta-analysis. J Dent. 2021;112:103765. doi: 10.1016/j.jdent.2021.103765. 34363890

[34] Espona-Noguera A, Živanić M, Smits E, et al. Unlocking novel anticancer strategies: bioactive hydrogels for local delivery of plasma-derived oxidants in an in ovo cancer model. Macromol Biosci. 2024;24(11):e2400213. doi: 10.1002/mabi.202400213. 38899954

[35] He H, Hao Y, Fan Y, et al. The interaction between innate immunity and oral microbiota in oral diseases. Expert Rev Clin Immunol. 2023;19(4):405–415. doi: 10.1080/1744666X.2023.2182291. 36803467

[36] Lamont RJ, Koo H, Hajishengallis G. The oral microbiota: dynamic communities and host interactions. Nat Rev Microbiol. 2018;16(12):745–759. doi: 10.1038/s41579-018-0089-x. 30301974 PMC6278837

[37] Sukmana BI, Saleh RO, Najim MA, et al. Oral microbiota and oral squamous cell carcinoma: A review of their relation and carcinogenic mechanisms. Front Oncol. 2024;14:1319777. doi: 10.3389/fonc.2024.1319777. 38375155 PMC10876296

[38] Giordano-Kelhoffer B, Lorca C, March Llanes J, et al. Oral microbiota, its equilibrium and implications in the pathophysiology of human diseases: a systematic review. Biomedicines. 2022;10(8):1803. doi: 10.3390/biomedicines10081803. 36009350 PMC9405223

[39] Newman KL, Kamada N. Pathogenic associations between oral and gastrointestinal diseases. Trends Mol Med. 2022;28(12):1030–1039. doi: 10.1016/j.molmed.2022.05.006. 35691866 PMC9691515

[40] Wen ZT, Huang X, Ellepola K, et al. *Lactobacilli* and human dental caries: more than mechanical retention. Microbiology (Reading). 2022;168(6):001196. doi: 10.1099/mic.0.001196. 35671222 PMC10233465

[41] Tortora SC, Agurto MG, Martello LA. The oral-gut-circulatory axis: from homeostasis to colon cancer. Front Cell Infect Microbiol. 2023;13:1289452. doi: 10.3389/fcimb.2023.1289452. 38029267 PMC10663299

[42] Yuan S, Fang C, Leng W-D, et al. Oral microbiota in the oral-genitourinary axis: identifying periodontitis as a potential risk of genitourinary cancers. Mil Med Res. 2021;8(1):54. doi: 10.1186/s40779-021-00344-1. 34588004 PMC8480014

[43] Jiang Y, Yin C, Mo J, et al. Recent progress in carbon dots for anti-pathogen applications in oral cavity. Front Cell Infect Microbiol. 2023;13:1251309. doi: 10.3389/fcimb.2023.1251309. 37780847 PMC10540312

[44] Sah AK, Dewangan M, Suresh PK. Potential of chitosan-based carrier for periodontal drug delivery. Colloids Surf B Biointerfaces. 2019;178:185–198. doi: 10.1016/j.colsurfb.2019.02.044. 30856588

[45] Duarte S, Kuo SP, Murata RM, et al. Air plasma effect on dental disinfection. Physics of Plasmas. 2011;18(7):073503. doi: 10.1063/1.3606486.

[46] Nima G, Harth-Chu E, Hiers RD, et al. Antibacterial efficacy of non-thermal atmospheric plasma against *Streptococcus mutans* biofilm grown on the surfaces of restorative resin composites. Sci Rep. 2021;11(1):23800. doi: 10.1038/s41598-021-03192-0. 34893687 PMC8664839

[47] Koban I, Geisel MH, Holtfreter B, et al. Synergistic ­effects of nonthermal plasma and disinfecting agents against dental biofilms in vitro. ISRN Dent. 2013;2013:573262. doi: 10.1155/2013/573262. 24159388 PMC3789417

[48] Hong Q, Dong X, Chen M, et al. Disinfection of *Streptococcus mutans* biofilm by a non-thermal atmospheric plasma brush. Jpn J Appl Phys. 2016;55(7S2):07LG02. doi: 10.7567/JJAP.55.07LG02.

[49] Park SR, Lee HW, Hong JW, et al. Enhancement of the killing effect of low-temperature plasma on *Streptococcus mutans* by combined treatment with gold nanoparticles. J Nanobiotechnol. 2014;12(1):29. doi: 10.1186/s12951-014-0029-5. PMC4237858 25104171

[50] Yang B, Chen J, Yu Q, et al. Oral bacterial deactivation using a low-temperature atmospheric argon plasma brush. J Dent. 2011;39(1):48–56. doi: 10.1016/j.jdent.2010.10.002. 20951184 PMC3010533

[51] Rupf S, Lehmann A, Hannig M, et al. Killing of adherent oral microbes by a non-thermal atmospheric plasma jet. J Med Microbiol. 2010;59(Pt 2):206–212. doi: 10.1099/jmm.0.013714-0. 19910483

[52] Blumhagen A, Singh P, Mustapha A, et al. Plasma deactivation of oral bacteria seeded on hydroxyapatite disks as tooth enamel analogue. Am J Dent. 2014;27(2):84–90. 25000666 PMC4090609

[53] Abonti TR, Kaku M, Kojima S, et al. Irradiation effects of low temperature multi gas plasma jet on oral bacteria. Dent Mater J. 2016;35(5):822–828. doi: 10.4012/dmj.2016-062. 27725521

[54] Li Y, Pan J, Ye G, et al. In vitro studies of the antimicrobial effect of non-thermal plasma-activated water as a novel mouthwash. Eur J Oral Sci. 2017;125(6):463–470. doi: 10.1111/eos.12374. 29024061

[55] Loesche WJ. Role of *Streptococcus mutans* in human dental decay. Microbiol Rev. 1986;50(4):353–380. doi: 10.1128/mr.50.4.353-380.1986. 3540569 PMC373078

[56] Lemos JA, Burne RA. A model of efficiency: stress tolerance by *Streptococcus mutans*. Microbiology (Reading). 2008;154(Pt 11):3247–3255. doi: 10.1099/mic.0.2008/023770-0. 18957579 PMC2627771

[57] Lemos JA, Palmer SR, Zeng L, et al. The biology of *Streptococcus mutans*. Microbiol Spectr. 2019;7(1):GPP3-0051-2018. doi: 10.1128/microbiolspec.GPP3-0051-2018. PMC6615571 30657107

[58] Park K-H, Nam S-H, Kim G-C, The Korean Society of Oral Health Science. Effects of combined treatment by glycyrrhiza uralensis extract and low-temperature plasma on *Streptococcus Mutans*. Korean Soc Oral Health Sci. 2022;10(3):74–79. doi: 10.33615/jkohs.2022.10.3.74.

[59] Tasaki T, Ohshima T, Usui E, et al. Plasma-treated water eliminates *Streptococcus mutans* in infected dentin model. Dent Mater J. 2017;36(4):422–428. doi: 10.4012/dmj.2016-358. 28367914

[60] Qiao D, Li Y, Pan J, et al. Effect of plasma activated water in caries prevention: the caries related biofilm inhibition effects and mechanisms. Plasma Chem Plasma Process. 2022;42(4):801–814. doi: 10.1007/s11090-022-10244-4.

[61] Milhan NVM, Chiappim W, Sampaio AdG, et al. Applications of plasma-activated water in dentistry: a review. Int J Mol Sci. 2022;23(8):4131. doi: 10.3390/ijms23084131. 35456947 PMC9029124

[62] Byun R, Nadkarni MA, Chhour K-L, et al. Quantitative analysis of diverse *Lactobacillus* species present in advanced dental caries. J Clin Microbiol. 2004;42(7):3128–3136. doi: 10.1128/JCM.42.7.3128-3136.2004. 15243071 PMC446321

[63] Caufield PW, Schön CN, Saraithong P, et al. Oral *Lactobacilli* and dental caries: a model for niche adaptation in humans. J Dent Res. 2015;94(9_suppl):110S–118S. doi: 10.1177/0022034515576052. 25758458 PMC4547204

[64] Bizhang M, Ellerbrock B, Preza D, et al. Detection of nine microorganisms from the initial carious root lesions using a TaqMan-based real-time PCR. Oral Dis. 2011;17(7):642–652. doi: 10.1111/j.1601-0825.2011.01815.x. 21605286

[65] Deng L, Li W, He Y, et al. Cross-kingdom interaction of *Candida albicans* and *Actinomyces viscosus* elevated cariogenic virulence. Arch Oral Biol. 2019;100:106–112. doi: 10.1016/j.archoralbio.2019.02.008. 30822704

[66] Dame-Teixeira N, Parolo CCF, Maltz M, et al. *Actinomyces* spp. Gene expression in root caries lesions. J Oral Microbiol. 2016;8(1):32383. doi: 10.3402/jom.v8.32383. 27640531 PMC5027334

[67] Liu D, Xiong Z, Du T, et al. Bacterial-killing effect of atmospheric pressure non-equilibrium plasma jet and oral mucosa response. J Huazhong Univ Sci Technolog Med Sci. 2011;31(6):852–856. doi: 10.1007/s11596-011-0690-y. 22173512

[68] Lima GdMG, Carta CFL, Borges AC, et al. Cold atmospheric pressure plasma is effective against *P. gingivalis* (HW24D-1) mature biofilms and non-genotoxic to oral cells. Appl Sci-Basel. 2022;12(14):7247. doi: 10.3390/app12147247.

[69] Carreiro AFP, Delben JA, Guedes S, et al. Low-temperature plasma on peri-implant-related biofilm and gingival tissue. J Periodontol. 2019;90(5):507–515. doi: 10.1002/JPER.18-0366. 30387879

[70] Lee J-Y, Kim K-H, Park S-Y, et al. The bactericidal effect of an atmospheric-pressure plasma jet on *Porphyromonas gingivalis* biofilms on sandblasted and acid-etched ­titanium discs. J Periodontal Implant Sci. 2019;49(5):319–329. doi: 10.5051/jpis.2019.49.5.319. 31681489 PMC6819695

[71] Hirano Y, Hayashi M, Tamura M, et al. Singlet oxygen generated by a new nonthermal atmospheric pressure air plasma device exerts a bactericidal effect on oral pathogens. J Oral Sci. 2019;61(4):521–525. doi: 10.2334/josnusd.18-0455. 31588099

[72] Hong Q, Sun H, Chen M, et al. Plasma treatment effects on destruction and recovery of *Porphyromonas gingivalis* biofilms. PLoS One. 2022;17(9):e0274523. doi: 10.1371/journal.pone.0274523. 36103549 PMC9473617

[73] Liguori A, Cochis A, Stancampiano A, et al. Cold atmospheric plasma treatment affects early bacterial adhesion and decontamination of soft reline palatal obturators. Clinical Plasma Medicine. 2017;7–8:36–45. doi: 10.1016/j.cpme.2017.08.001.

[74] Jungbauer G, Favaro L, Müller S, et al. The in-vitro activity of a cold atmospheric plasma device utilizing ambient air against bacteria and biofilms associated with periodontal or peri-implant diseases. Antibiotics (Basel). 2022;11(6):752. doi: 10.3390/antibiotics11060752. 35740158 PMC9219831

[75] Shi Q, Song K, Zhou X, et al. Effects of non-equilibrium plasma in the treatment of ligature-induced peri-implantitis. J Clin Periodontol. 2015;42(5):478–487. doi: 10.1111/jcpe.12403. 25867215

[76] Bodet C, Chandad F, Grenier D. Potentiel pathogénique de *Porphyromonas gingivalis*, *Treponema denticola* et *Tannerella forsythia*, le complexe bactérien rouge associé à la parodontite. Pathologie Biologie. 2007;55(3–4):154–162. doi: 10.1016/j.patbio.2006.07.045. 17049750

[77] Bostanci N, Belibasakis GN. *Porphyromonas gingivalis*: an invasive and evasive opportunistic oral pathogen. FEMS Microbiol Lett. 2012;333(1):1–9. doi: 10.1111/j.1574-6968.2012.02579.x. 22530835

[78] Mysak J, Podzimek S, Sommerova P, et al. *Porphyromonas gingivalis*: major periodontopathic pathogen overview. J Immunol Res. 2014;2014:e476068–8. doi: 10.1155/2014/476068. PMC3984870 24741603

[79] Lima GdMG, Borges AC, Nishime TMC, et al. Cold atmospheric plasma jet as a possible adjuvant therapy for periodontal disease. Molecules. 2021;26(18):5590. doi: 10.3390/molecules26185590. 34577061 PMC8470429

[80] Henderson B, Ward JM, Ready D. *Aggregatibacter* (*Actinobacillus*) *actinomycetemcomitans*: a triple A* periodontopathogen? Periodontol 2000. 2010;54(1):78–105. doi: 10.1111/j.1600-0757.2009.00331.x. 20712635

[81] Fine DH, Markowitz K, Furgang D, et al. *Aggregatibacter actinomycetemcomitans* as an early colonizer of oral tissues: epithelium as a reservoir? J Clin Microbiol. 2010;48(12):4464–4473. doi: 10.1128/JCM.00964-10. 20881174 PMC3008435

[82] Åberg CH, Kelk P, Johansson A. *Aggregatibacter actinomycetemcomitans*: virulence of its leukotoxin and association with aggressive periodontitis. Virulence. 2015;6(3):188–195. doi: 10.4161/21505594.2014.982428. 25494963 PMC4601274

[83] Mai-Prochnow A, Clauson M, Hong J, et al. Gram positive and Gram negative bacteria differ in their sensitivity to cold plasma. Sci Rep. 2016;6(1):38610. doi: 10.1038/srep38610. 27934958 PMC5146927

[84] de Andrade KQ, Almeida-da-Silva CLC, Coutinho-Silva R. Immunological pathways triggered by *Porphyromonas gingivalis* and *Fusobacterium nucleatum*: therapeutic possibilities? Mediators Inflamm. 2019;2019:e7241312–20. doi: 10.1155/2019/7241312. PMC6612971 31341421

[85] Socransky S. S, Haffajee A. D, Cugini M. A, et al. Microbial complexes in subgingival plaque. J Clin Periodontol. 1998;25(2):134–144. doi: 10.1111/j.1600-051X.1998.tb02419.x. 9495612

[86] Polak D, Wilensky A, Shapira L, et al. Mouse model of experimental periodontitis induced by *Porphyromonas gingivalis*/*Fusobacterium nucleatum* infection: bone loss and host response. J Clin Periodontol. 2009;36(5):406–410. doi: 10.1111/j.1600-051X.2009.01393.x. 19419440

[87] Sharma A, Inagaki S, Honma K, et al. *Tannerella forsythia*-induced alveolar bone loss in mice involves leucine-rich-repeat BspA protein. J Dent Res. 2005;84(5):462–467. doi: 10.1177/154405910508400512. 15840784

[88] Jusko M, Potempa J, Karim AY, et al. A metalloproteinase karilysin present in the majority of *Tannerella forsythia* isolates inhibits all pathways of the complement system. J Immunol. 2012;188(5):2338–2349. doi: 10.4049/jimmunol.1101240. 22287711 PMC3288752

[89] Lee J-Y, Jung Y-J, Jun H-K, et al. Pathogenic potential of *Tannerella forsythia* enolase. Mol Oral Microbiol. 2016;31(2):189–203. doi: 10.1111/omi.12115. 26172848

[90] Jung Y-J, Choi Y-J, An S-J, et al. *Tannerella forsythia* GroEL induces inflammatory bone resorption and synergizes with interleukin-17. Mol Oral Microbiol. 2017;32(4):301–313. doi: 10.1111/omi.12172. 27484636

[91] Yamanaka T, Furukawa T, Matsumoto-Mashimo C, et al. Gene expression profile and pathogenicity of biofilm-forming *Prevotella intermedia* strain 17. BMC Microbiol. 2009;9(1):11. doi: 10.1186/1471-2180-9-11. 19146705 PMC2633007

[92] Kornman KS, Loesche WJ. Effects of estradiol and progesterone on *Bacteroides melaninogenicus* and *Bacteroides gingivalis*. Infect Immun. 1982;35(1):256–263. doi: 10.1128/iai.35.1.256-263.1982. 6119293 PMC351023

[93] Könönen E, Fteita D, Gursoy UK, et al. Prevotella species as oral residents and infectious agents with potential impact on systemic conditions. J Oral Microbiol. 2022;14(1):2079814. doi: 10.1080/20002297.2022.2079814. 36393976 PMC9662046

[94] Eggers B, Marciniak J, Deschner J, et al. Cold atmospheric plasma promotes regeneration-associated cell functions of murine cementoblasts in vitro. Int J Mol Sci. 2021;22(10):5280. doi: 10.3390/ijms22105280. 34067898 PMC8156616

[95] Eggers B, Stope MB, Marciniak J, et al. Modulation of inflammatory responses by a non-invasive physical plasma jet during gingival wound healing. CELLS. 2022;11(17):2740. doi: 10.3390/cells11172740. 36078148 PMC9454534

[96] Miletic M, Mojsilovic S, Dordevic IO, et al. Effects of non-thermal atmospheric plasma on human periodontal ligament mesenchymal stem cells. J Phys D-Appl Phys. 2013;46(34):345401. doi: 10.1088/0022-3727/46/34/345401.

[97] Theinkom F, Singer L, Cieplik F, et al. Antibacterial efficacy of cold atmospheric plasma against *Enterococcus faecalis* planktonic cultures and biofilms in vitro. PLoS One. 2019;14(11):e0223925. doi: 10.1371/journal.pone.0223925. 31770390 PMC6879142

[98] Cao Y, Yang P, Lu X, et al. Efficacy of atmospheric pressure plasma as an antibacterial agent against *Enterococcus Faecalis* in vitro. Plasma Sci Technol. 2011;13(1):93–98. doi: 10.1088/1009-0630/13/1/19.

[99] Chang Y-T, Chen G. Oral bacterial inactivation using a novel low-temperature atmospheric-pressure plasma device. J Dent Sci. 2016;11(1):65–71. doi: 10.1016/j.jds.2014.03.007. 30894948 PMC6395194

[100] Pan J, Sun K, Liang Y, et al. Cold plasma therapy of a tooth root canal infected with *Enterococcus faecalis* biofilms in vitro. J Endod. 2013;39(1):105–110. doi: 10.1016/j.joen.2012.08.017. 23228267

[101] Kaya BÜ, Kececi AD, Güldaş HE, et al. Efficacy of endodontic applications of ozone and low-temperature atmospheric pressure plasma on root canals infected with *Enterococcus faecalis*. Lett Appl Microbiol. 2014;58(1):8–15. doi: 10.1111/lam.12148. 23980743

[102] Herbst SR, Hertel M, Ballout H, et al. Bactericidal efficacy of cold plasma at different depths of infected root canals in vitro. Open Dent J. 2015;9(1):486–491. doi: 10.2174/1874210601509010486. 26962378 PMC4768658

[103] Hüfner A, Steffen H, Holtfreter B, et al. Effects of non-thermal atmospheric pressure plasma and sodium hypochlorite solution on *Enterococcus faecalis* biofilm: an investigation in extracted teeth. Plasma Process Polym. 2017;14(3):1600064. doi: 10.1002/ppap.201600064.

[104] Du T, Shi Q, Shen Y, et al. Effect of modified nonequilibrium plasma with chlorhexidine digluconate against endodontic biofilms in vitro. J Endod. 2013;39(11):1438–1443. doi: 10.1016/j.joen.2013.06.027. 24139270

[105] Zhou X-C, Li Y-L, Liu D-X, et al. Bactericidal effect of plasma jet with helium flowing through 3% hydrogen peroxide against *Enterococcus faecalis*. Exp Ther Med. 2016;12(5):3073–3077. doi: 10.3892/etm.2016.3726. 27882119 PMC5103749

[106] Tschang C-YT, Thoma M. Biofilm inactivation by synergistic treatment of atmospheric pressure plasma and chelating agents. Clin Plasma Med. 2019;15:100091. doi: 10.1016/j.cpme.2019.100091.

[107] Armand A, Khani M, Asnaashari M, et al. Comparison study of root canal disinfection by cold plasma jet and photodynamic therapy. Photodiagnosis Photodyn Ther. 2019;26:327–333. doi: 10.1016/j.pdpdt.2019.04.023. 31026615

[108] Ballout H, Hertel M, Doehring J, et al. Effects of plasma jet, dielectric barrier discharge, photodynamic therapy and sodium hypochlorite on infected curved root canals. J Biophotonics. 2018;11(3):e201700186. doi: 10.1002/jbio.201700186. 29024574

[109] Li Y, Sun K, Ye G, et al. Evaluation of cold plasma treatment and safety in disinfecting 3-week root canal *Enterococcus faecalis* biofilm in vitro. J Endod. 2015;41(8):1325–1330. doi: 10.1016/j.joen.2014.10.020. 26027875

[110] Yao Y, Song K, Chen H, et al. In vitro and in vivo research of atmosphere pressure nonequilibrium plasmas on root canal disinfection: implication for alternative strategy for irrigation. Clin Oral Investig. 2021;25(10):5833–5842. doi: 10.1007/s00784-021-03888-7. 33763712

[111] Kim YM, Choi BBR, Park SR, et al. Antimicrobial effect of low temperature atmospheric plasma against oral pathogens. Intern J Oral Biol. 2015;40(4):167–173. doi: 10.11620/IJOB.2015.40.4.167.

[112] Pu Q-K, Liu S-J, Huang H, et al. Sterilization effect of an atmospheric low temperature plasma jet on *Candida albicans* biofilm. Sichuan Da Xue Xue Bao Yi Xue Ban. 2019;50(3):339–343. doi: 10.13464/j.scuxbyxb.2019.03.010. 31631600

[113] Nishime TMC, Borges AC, Koga-Ito CY, et al. Non-thermal atmospheric pressure plasma jet applied to inactivation of different microorganisms. Surface & Coatings Technol. 2017;312:19–24. doi: 10.1016/j.surfcoat.2016.07.076.

[114] Pan H, Wang G, Pan J, et al. Cold plasma-induced surface modification of heat-polymerized acrylic resin and prevention of early adherence of *Candida albicans*. Dent Mater J. 2015;34(4):529–536. doi: 10.4012/dmj.2015-035. 26235720

[115] Borges AC, Lima GdMG, Nishime TMC, et al. Amplitude-modulated cold atmospheric pressure plasma jet for treatment of oral candidiasis: in vivo study. PLoS One. 2018;13(6):e0199832. doi: 10.1371/journal.pone.0199832. 29949638 PMC6021106

[116] Leite LDP, Oliveira MACd, Vegian MRdC, et al. Effect of cold atmospheric plasma jet associated to polyene antifungals on *Candida albicans* biofilms. Molecules. 2021;26(19):5815. doi: 10.3390/molecules26195815. 34641359 PMC8510435

[117] Preissner S, Kastner I, Schütte E, et al. Adjuvant antifungal therapy using tissue tolerable plasma on oral mucosa and removable dentures in oral candidiasis patients: a randomised double-blinded split-mouth pilot study. Mycoses. 2016;59(7):467–475. doi: 10.1111/myc.12495. 26932256

[118] Matthes R, Jablonowski L, Koban I, et al. In vitro treatment of *Candida albicans* biofilms on denture base material with volume dielectric barrier discharge plasma (VDBD) compared with common chemical antiseptics. Clin Oral Investig. 2015;19(9):2319–2326. doi: 10.1007/s00784-015-1463-y. 25898894

[119] Alekseev O, Donovan K, Limonnik V, et al. Nonthermal Dielectric Barrier Discharge (DBD) plasma suppresses herpes simplex virus type 1 (HSV-1) replication in corneal epithelium. Transl Vis Sci Technol. 2014;3(2):2. doi: 10.1167/tvst.3.2.2. PMC3969218 24757592

[120] Bunz O, Mese K, Funk C, et al. Cold atmospheric plasma as antiviral therapy—Effect on human herpes simplex virus type 1. J Gen Virol. 2020;101(2):208–215. doi: 10.1099/jgv.0.001382. 31961788 PMC7414428

[121] Elashiry MM, Bergeron BE, Tay FR. Enterococcus faecalis in secondary apical periodontitis: mechanisms of bacterial survival and disease persistence. Microb Pathog. 2023;183:106337. doi: 10.1016/j.micpath.2023.106337. 37683835

[122] Haapasalo M, Orstavik D. In vitro infection and disinfection of dentinal tubules. J Dent Res. 1987;66(8):1375–1379. doi: 10.1177/00220345870660081801. 3114347

[123] Liu Z, Xie Z, Li W, et al. Photodynamic immunotherapy of cancers based on nanotechnology: recent advances and future challenges. J Nanobiotechnol. 2021;19(1):160. doi: 10.1186/s12951-021-00903-7. PMC8164771 34051801

[124] Alves-Silva EG, Arruda-Vasconcelos R, Louzada LM, et al. Effect of antimicrobial photodynamic therapy on the reduction of bacteria and virulence factors in teeth with primary endodontic infection. Photodiagnosis Photodyn Ther. 2023;41:103292. doi: 10.1016/j.pdpdt.2023.103292. 36681260

[125] Mayer FL, Wilson D, Hube B. *Candida albicans* pathogenicity mechanisms. Virulence. 2013;4(2):119–128. doi: 10.4161/viru.22913. 23302789 PMC3654610

[126] Pappas PG, Kauffman CA, Andes D, et al. Clinical practice guidelines for the management of candidiasis: 2009 update by the Infectious Diseases Society of America. Clin Infect Dis. 2009;48(5):503–535. doi: 10.1086/596757. 19191635 PMC7294538

[127] Hebecker B, Naglik JR, Hube B, et al. Pathogenicity mechanisms and host response during oral *Candida albicans* infections. Expert Rev Anti Infect Ther. 2014;12(7):867–879. doi: 10.1586/14787210.2014.916210. 24803204

[128] Yang Z, Zhang S, Ji N, et al. The evil companion of OSCC: *Candida albicans*. Oral Dis. 2024;30(4):1873–1886. doi: 10.1111/odi.14700. 37530513

[129] Garcia-Cuesta C, Sarrion-Pérez M-G, Bagán JV. Current treatment of oral candidiasis: a literature review. J Clin Exp Dent. 2014;6(5):e576–e582. doi: 10.4317/jced.51798. 25674329 PMC4312689

[130] Perlin DS, Rautemaa-Richardson R, Alastruey-Izquierdo A. The global problem of antifungal resistance: prevalence, mechanisms, and management. Lancet Infect Dis. 2017;17(12):e383–e392. doi: 10.1016/S1473-3099(17)30316-X. 28774698

[131] Menendez CM, Carr DJJ. Defining nervous system susceptibility during acute and latent herpes simplex virus-1 infection. J Neuroimmunol. 2017;308:43–49. doi: 10.1016/j.jneuroim.2017.02.020. 28302316 PMC5474347

[132] Brown JC. Herpes simplex virus latency: the DNA repair-centered pathway. Adv Virol. 2017;2017:7028194–7028196. doi: 10.1155/2017/7028194. 28255301 PMC5309397

[133] Mohamed H, Nayak G, Rendine N, et al. Non-thermal plasma as a novel strategy for treating or preventing viral infection and associated disease. Front Phys. 2021;9:683118. doi: 10.3389/fphy.2021.683118.

[134] Filipić A, Gutierrez-Aguirre I, Primc G, et al. Cold plasma, a new hope in the field of virus inactivation. Trends Biotechnol. 2020;38(11):1278–1291. doi: 10.1016/j.tibtech.2020.04.003. 32418663 PMC7164895

[135] Bowen WH, Burne RA, Wu H, et al. Oral biofilms: pathogens, matrix, and polymicrobial interactions in microenvironments. Trends Microbiol. 2018;26(3):229–242. doi: 10.1016/j.tim.2017.09.008. 29097091 PMC5834367

[136] Marsh PD, Zaura E. Dental biofilm: ecological interactions in health and disease. J Clin Periodontol. 2017;44 Suppl 18(S18):S12–S22. doi: 10.1111/jcpe.12679. 28266111

[137] Blanc V, Isabal S, Sánchez MC, et al. Characterization and application of a flow system for in vitro multispecies oral biofilm formation. J Periodontal Res. 2014;49(3):323–332. doi: 10.1111/jre.12110. 23815431

[138] Sedlacek MJ, Walker C. Antibiotic resistance in an in vitro subgingival biofilm model. Oral Microbiol Immunol. 2007;22(5):333–339. doi: 10.1111/j.1399-302X.2007.00366.x. 17803631 PMC2040071

[139] Rao Y, Shang W, Yang Y, et al. Fighting mixed-species microbial biofilms with cold atmospheric plasma. Front Microbiol. 2020;11:1000. doi: 10.3389/fmicb.2020.01000. 32508796 PMC7251026

[140] Figueira LW, Panariello BHD, Koga-Ito CY, et al. Low-temperature plasma as an approach for inhibiting a multi-species cariogenic biofilm. Applied Sciences-Basel. 2021;11(2):570. doi: 10.3390/app11020570.

[141] Xiong Z, Du T, Lu X, et al. How deep can plasma penetrate into a biofilm? Appl Phys Lett. 2011;98(22):221503. doi: 10.1063/1.3597622.

[142] Pei X, Lu X, Liu J, et al. Inactivation of a 25.5 µm *Enterococcus faecalis* biofilm by a room-temperature, battery-operated, handheld air plasma jet. J Phys D: Appl Phys. 2012;45(16):165205. doi: 10.1088/0022-3727/45/16/165205.

[143] Matthes R, Bender C, Schlüter R, et al. Antimicrobial efficacy of two surface barrier discharges with air ­plasma against in vitro biofilms. PLoS One. 2013;8(7):e70462. doi: 10.1371/journal.pone.0070462. 23894661 PMC3722131

[144] Puac N, Miletic M, Mojovic M, et al. Sterilization of bacteria suspensions and identification of radicals deposited during plasma treatment. Open Chemistry. 2015;13(1):332–338. doi: 10.1515/chem-2015-0041.

[145] Tang X, Shi Q, Zhang Z, et al. Immediate and sustained killing effects of atmospheric-pressure plasma on young and mature biofilms of *Enterococcus faecalis*. AIP Advances. 2021;11(5):055118. doi: 10.1063/5.0051305.

[146] Hong Q, Dong X, Chen M, et al. An in vitro and in vivo study of plasma treatment effects on oral biofilms. J Oral Microbiol. 2019;11(1):1603524. doi: 10.1080/20002297.2019.1603524. 31069019 PMC6493255

[147] Ziuzina D, Boehm D, Patil S, et al. Cold plasma inactivation of bacterial biofilms and reduction of quorum sensing regulated virulence factors. PLoS One. 2015;10(9):e0138209. doi: 10.1371/journal.pone.0138209. 26390435 PMC4577073

[148] Flynn PB, Busetti A, Wielogorska E, et al. Non-thermal plasma exposure rapidly attenuates bacterial AHL-dependent quorum sensing and virulence. Sci Rep. 2016;6(1):26320. doi: 10.1038/srep26320. 27242335 PMC4886528

[149] Czapka T, Maliszewska I, Olesiak-Bańska J. Influence of atmospheric pressure non-thermal plasma on inactivation of biofilm cells. Plasma Chem Plasma Process. 2018;38(6):1181–1197. doi: 10.1007/s11090-018-9925-z.

[150] Rovetta-Nogueira SdM, Borges AC, Oliveira Filho M D, et al. Helium cold atmospheric plasma causes morphological and biochemical alterations in *Candida albicans* cells. Molecules. 2023;28(23):7919. doi: 10.3390/molecules28237919. 38067648 PMC10707892

[151] Lunder M, Dahle S, Fink R. Cold atmospheric plasma for surface disinfection: a promising weapon against deleterious meticillin-resistant *Staphylococcus aureus* biofilms. J Hosp Infect. 2024;143:64–75. doi: 10.1016/j.jhin.2023.10.014. 37939884

[152] Navabsafa N, Ghomi H, Nikkhah M, et al. Effect of BCD plasma on a bacteria cell membrane. Plasma Sci Technol. 2013;15(7):685–689. doi: 10.1088/1009-0630/15/7/15.

[153] Zhao Y, Shao L, Jia L, et al. Inactivation effects, kinetics and mechanisms of air- and nitrogen-based cold atmospheric plasma on *Pseudomonas aeruginosa*. Innov Food Sci Emerg Technol. 2022;79:103051. doi: 10.1016/j.ifset.2022.103051.

[154] Soler-Arango J, Figoli C, Muraca G, et al. The *Pseudomonas aeruginosa* biofilm matrix and cells are drastically impacted by gas discharge plasma treatment: a comprehensive model explaining plasma-mediated biofilm eradication. PLoS One. 2019;14(6):e0216817. doi: 10.1371/journal.pone.0216817. 31233528 PMC6590783

[155] Joshi SG, Cooper M, Yost A, et al. Nonthermal dielectric-barrier discharge plasma-induced inactivation involves oxidative DNA damage and membrane lipid peroxidation in *Escherichia coli*. Antimicrob Agents Chemother. 2011;55(3):1053–1062. doi: 10.1128/AAC.01002-10. 21199923 PMC3067084

[156] Kobzev EN, Kireev GV, Rakitskii YA, et al. Effect of cold plasma on the *E. coli* cell wall and plasma membrane. Appl Biochem Microbiol. 2013;49(2):144–149. doi: 10.1134/S0003683813020063. 23795475

[157] Zhang L, Zhang D, Guo Y, et al. Surface decontamination by atmospheric pressure plasma jet: key ­biological processes. J Phys D: Appl Phys. 2022;55(42):425203. doi: 10.1088/1361-6463/ac8432.

[158] Kreuzer KN. DNA damage responses in prokaryotes: regulating gene expression, modulating growth patterns, and manipulating replication forks. Cold Spring Harb Perspect Biol. 2013;5(11):a012674. doi: 10.1101/cshperspect.a012674. 24097899 PMC3809575

[159] Sharma A, Collins G, Pruden A. Differential gene ­expression in *Escherichia coli* following exposure to nonthermal atmospheric pressure plasma. J Appl Microbiol. 2009;107(5):1440–1449. doi: 10.1111/j.1365-2672.2009.04323.x. 19426273

[160] Ebrahimi-Shaghaghi F, Atyabi S-M, Razzaghi-Abyaneh M. Plasma-based strategy for inhibiting *Candida albicans* growth and *CaMCA1* gene expression in vitro and reducing fungal pathogenicity in a murine model of vulvovaginal candidiasis. Med Mycol. 2021a;60(1):myab067. doi: 10.1093/mmy/myab067. 34694384

[161] Ebrahimi-Shaghaghi F, Noormohammadi Z, Atyabi S-M, et al. Inhibitory effects of cold atmospheric plasma on the growth, virulence factors and *HSP90* gene expression in *Candida albicans*. Arch Biochem Biophys. 2021b;700:108772. doi: 10.1016/j.abb.2021.108772. 33485850

[162] Barkhade T, Nigam K, Ravi G, et al. Plasma sterilization for bacterial inactivation: studies on probable mechanisms and biochemical actions. Plasma Chem Plasma Process. 2024;44(1):429–454. doi: 10.1007/s11090-023-10429-5.

[163] Winter T, Winter J, Polak M, et al. Characterization of the global impact of low temperature gas plasma on vegetative microorganisms. Proteomics. 2011;11(17):3518–3530. doi: 10.1002/pmic.201000637. 21751354

[164] Guo L, Xu R, Gou L, et al. Mechanism of virus inactivation by cold atmospheric-pressure plasma and plasma-activated water. Appl Environ Microbiol. 2018;84(17):e00726. doi: 10.1128/AEM.00726-18. 29915117 PMC6102979

[165] Graves DB. The emerging role of reactive oxygen and nitrogen species in redox biology and some implications for plasma applications to medicine and biology. J Phys D: Appl Phys. 2012;45(26):263001. doi: 10.1088/0022-3727/45/26/263001.

[166] Laroussi M, Leipold F. Evaluation of the roles of reactive species, heat, and UV radiation in the inactivation of bacterial cells by air plasmas at atmospheric pressure. Int J Mass Spectrom. 2004;233(1–3):81–86. doi: 10.1016/j.ijms.2003.11.016.

[167] Deng X, Shi J, Kong MG. Physical mechanisms of inactivation of *Bacillus subtilis* spores using cold atmospheric plasmas. IEEE Trans Plasma Sci. 2006;34(4):1310–1316. doi: 10.1109/TPS.2006.877739.

[168] Lackmann J-W, Schneider S, Edengeiser E, et al. Photons and particles emitted from cold atmospheric-pressure plasma inactivate bacteria and biomolecules independently and synergistically. J R Soc Interface. 2013;10(89):20130591. doi: 10.1098/rsif.2013.0591. 24068175 PMC3808546

[169] Lackmann J-W, Bandow JE. Inactivation of microbes and macromolecules by atmospheric-pressure plasma jets. Appl Microbiol Biotechnol. 2014;98(14):6205–6213. doi: 10.1007/s00253-014-5781-9. 24841116

[170] Salgado BAB, Fabbri S, Dickenson A, et al. Surface barrier discharges for *Escherichia coli* biofilm inactivation: modes of action and the importance of UV radiation. PLoS One. 2021;16(3):e0247589. doi: 10.1371/journal.pone.0247589. 33730103 PMC7968650

[171] Murakami T, Niemi K, Gans T, et al. Chemical kinetics and reactive species in atmospheric pressure helium–oxygen plasmas with humid-air impurities. Plasma Sources Sci Technol. 2012;22(1):015003. doi: 10.1088/0963-0252/22/1/015003.

[172] Graves DB. Reactive species from cold atmospheric plasma: implications for cancer therapy. Plasma Processes & Polymers. 2014;11(12):1120–1127. doi: 10.1002/ppap.201400068.

[173] Boekema B, Stoop M, Vlig M, et al. Antibacterial and safety tests of a flexible cold atmospheric plasma device for the stimulation of wound healing. Appl Microbiol Biotechnol. 2021;105(5):2057–2070. doi: 10.1007/s00253-021-11166-5. 33587156 PMC7906937

[174] Oliveira MACd, Lima GdMG, Nishime TMC, et al. Inhibitory effect of cold atmospheric plasma on chronic wound-related multispecies biofilms. Appl Sci. 2021;11(12):5441. doi: 10.3390/app11125441.

[175] Jha N, Ryu JJ, Choi EH, et al. Generation and role of reactive oxygen and nitrogen species induced by ­plasma, lasers, chemical agents, and other systems in ­dentistry. Oxid Med Cell Longev. 2017;2017(1):e7542540. doi: 10.1155/2017/7542540. PMC5674515 29204250

[176] Lu X, Bruggeman PJ, Reuter S, et al. Grand challenges in low temperature plasmas. Front Phys. 2022;10:1040658. doi: 10.3389/fphy.2022.1040658.

[177] Lin A, Biscop E, Gorbanev Y, et al. Toward defining plasma treatment dose: the role of plasma treatment energy of pulsed-dielectric barrier discharge in dictating in vitro biological responses. Plasma Processes & Polymers. 2022;19(3):e2100151. doi: 10.1002/ppap.202100151.

[178] von Woedtke T, Reuter S, Masur K, et al. Plasmas for medicine. Phys Reports-Rev Section of Phys Lett. 2013;530(4):291–320. doi: 10.1016/j.physrep.2013.05.005.

[179] Cheng H, Luo J, Song K, et al. On the dose of plasma medicine: plasma-activated medium (PAM) and its effect on cell viability. Physics of Plasmas. 2022;29(6):063506. doi: 10.1063/5.0089357.

[180] Koban I, Matthes R, Hübner N-O, et al. Treatment of *Candida albicans* biofilms with low-temperature plasma induced by dielectric barrier discharge and atmospheric pressure plasma jet. New J Phys. 2010;12(7):073039. doi: 10.1088/1367-2630/12/7/073039.

